# Adaptive Block Relative Edge Density (ABRED): An Interpretable Feature for Tunnel Surrounding Rock Fracture Identification

**DOI:** 10.3390/s26175488

**Published:** 2026-08-29

**Authors:** Bolin Jiang, Yunxue Luo, Shanshan Wu

**Affiliations:** 1 Office of Scientific Research, Chongqing Polytechnic University of Engineering, Chongqing 402260, China; 2 School of Civil Engineering, Chongqing Jiaotong University, Chongqing 400074, China; luoyunxue@outlook.com; 3 School of Railways and Architecture, Chongqing Vocational College of Public Transportation, Chongqing 402247, China; shanshanwu84@163.com

**Keywords:** edge concentration, context-aware region partitioning, normalized local descriptor, visual categorization, structural pattern characterization

## Abstract

**Highlights:**

**What are the main findings?**

- The ABRED feature framework addresses three key challenges, including spatial inconsistency, multiplicative lighting effects, and multi-scale variability, by integrating gradient-based, variance-driven quadtree decomposition; a relative density metric computed from eight surrounding pixels; and adaptive multi-scale smoothing.
- Evaluated on a practical tunnel surrounding rock dataset comprising 1200 real-world images, ABRED attains an accuracy of 87.6%, surpassing conventional fixed-block approaches as well as established texture descriptors including LBP, HOG, and Gabor filters.

**What are the implications of the main findings?**

- ABRED features a compact, interpretable architecture that supports efficient, low-latency classification on standard CPU platforms, making it well-suited for periodic tunnel structural safety assessment in field operations where computational resources may be limited.
- This research underscores the effectiveness of incorporating domain-grounded physical constraints into manually engineered features, establishing a robust, transparent (“white box”) methodology that serves as a reliable alternative to black box deep learning models in high-stakes engineering applications, particularly when interpretability and computational efficiency are prioritized.

**Abstract:**

Fracture density estimation from tunnel face imagery is a critical step in rock mass classification and quantification of fragmentation severity. However, traditional texture descriptors have three inherent drawbacks: fixed spatial partitioning, sensitivity to illumination, and lack of modeling of local contrast variations. To overcome these problems, we propose the Adaptive Block Relative Edge Density (ABRED) feature as a physically motivated, interpretable design for geological image interpretation. A quadtree decomposition guided by local gradient variance dynamically adjusts region granulation to be finer in areas with denser fracture and coarser otherwise. ABRED directly calculates relative edge density, where relative edge density is defined as the ratio of a block’s normalized edge count to the mean edge count of its eight neighboring blocks, demonstrating empirical robustness to illumination variations in our tests. Multi-scale representation is performed by aggregating Gaussian blur edge density maps on gradually blurring scales. Compared to fixed-grid competitors and handcrafted features, including LBP, HOG, and Gabor, ABRED achieves an overall classification performance of 87.6% on an operational, real-world dataset of 1200 tunnel surrounding rock images annotated with three unique fracture severity classes, significantly outperforming baseline fixed-grid methods and handcrafted feature approaches. Ablation studies confirm each component contributes individually, while PCA-based embedding results in well-separated, class-discriminative clusters. Importantly, by incorporating domain-specific physical information directly into its feature engineering—the opposite of data-driven black box optimization—ABRED achieves performance comparable to recent deep learning approaches on our specific dataset (87.6% vs. 88–91% reported for CNN-based methods under similar conditions), while requiring substantially lower computational overhead and offering full interpretability. We note, however, that deep learning models may achieve superior accuracy given substantially larger training datasets. The advantage of ABRED lies in its efficiency, transparency, and suitability for CPU-based field deployment where resources are constrained.

## 1. Introduction

Fracturing state of the rock mass at a tunnel face is a direct measure of structural weakness and severity of fracturing; reliable discrimination of three distinct fracture regimes of interest, including low density (intact/weak fractured zones), medium density (moderate fractured zones), and high density (strongly broken/fault-parallel zones), is the crucial link in informing the correct choice of ground support schemes and protecting the works. Traditionally applied fracture assessment methods, including borehole cores or field-observed mapping, yield highly spatially sparse data, are extremely labor-intensive and time-consuming to gather, and remain fundamentally discontinuous [1]. Vision-based methods using imagery at tunnel faces are a faster, non-invasive, cost-effective alternative which, through quantitative analysis of joint spacing, trace continuity, and associated geometric measures, estimate rock quality [2]. However, practical application faces significant challenges: real tunnel photographs are frequently degraded by non-uniform illumination, heterogeneous texture scales, aero-erosion, and humidity seepage, leading to a high degree of confounding within patterns and the need for highly robust and informative features with strong class-separating power.

Recent investigations on characterization of rock fractures in a tunnel setting can be broadly divided into three classes of technical approaches: ad hoc (i.e., manually engineered) texture features, purely data-driven deep architectures, and hybrid representations derived from physics, especially those that utilize edge density as a proxy for structural information [3]. In the realm of the former, researchers have proposed and reused a comprehensive set of handcrafted descriptors for quantifying surface inhomogeneity and fracture topology. A broadly accepted approach uses multi-scale feature fusion to enhance sensitivity in detection to fine-grained fissures by pooling information at different spatial granularities [4]. Another direction relies on hierarchical semantic segmentation of cracks for accurate identification and analysis of crack distributions in tunnel lining construction [5]. Convolutional architectures have also been utilized for mapping cracks at high resolution and for short-term propagation modelling at the pixel level [6]. However, traditional texture methods such as LBP, Histograms of Oriented Gradients (HOG), and Gabor-based filtering are based on fixed, isotropic sampling grids that make assumptions contradicting inherent spatial heterogeneities in fracture networks; this makes them unable to capture local structural intricacy. Even worse, these methods have no intrinsic inbuilt means to remove illumination-induced deformations or to describe local intensity contrast geometry.

On the other hand, DL-based solutions have achieved promising progress in terms of accuracy and automation. Examples include a specialized CNN model optimized for crack severity grading from shield tunnel lining images [7], DL-based encoders on the 3D point cloud to reconstruct discontinuity geometry and orientation in space [8], the integration of unsupervised clustering with the multi-modal sensor data for rock mass integrity inference [9], a back-propagation network to classify surrounding rock conditions for plateau tunnels [10], the stochastic Discrete Fracture Network (DFN) for construction quality assessment [11], or the extensive review and analysis for vision-based joint/failure identification for challenging mountainous tunnels [12]. Even though these models achieve empirical success, practical limitations remain: they require extensive labeled training samples, massive GPU computing and long iteration times—and critically—serve as black box models. These black box models hinder domain experts’ validation of the models, fault diagnosis, and deployment at edge devices that might not be well endowed with computing resources for field monitoring systems.

As for edge density-based methodologies, empirical evidence continuously advocates for edge concentration as a robust and physically intuitively meaningful descriptor for fracture analysis. However, all current implementations face three essential shortfalls: (i) reliance on static non-responsive spatial partitioning schemes; (ii) susceptibility of raw edge counts to ambient lighting variability; and (iii) insufficient leverage on contextual contrast, particularly on relative intensity variations across adjacent regions. For example, effective segmentation performance has been obtained on both tunnel lining surfaces as well as generic crack datasets [13], but the underlying edge features lack adaptive normalizations for dealing with illumination gradients, resulting in decreased performance under non-uniformly illuminated conditions. Similarly, rock mass classification using clustering or shallow N feature models [14] typically compute statistics over windows and rigid globally defined image domains, entirely ignoring that the spatial variance in fracture density across rock mass. Here, being inherently local and neighborhood dependent is crucial for confident discrimination on geologically variable scenarios.

Although recent progress has advanced fracture analysis significantly, three fundamental limitations persist across prevailing methods: (i) Rigid spatial partitioning where traditional descriptors (e.g., LBP, HOG, most edge density-based methods) enforce strict block dimensions that do not consider intrinsic spatial variation in fracture distribution. Continuum blocks of intact rock benefit from less grainy sampling to preserve statistical fidelity, while high-energy fracture zones require finer detail preservation—something not possible with fixed-grid schemes. (ii) Illumination-dependent methods are naturally sensitive in field environments in tunnels. (iii) Lack of context modeling: Previous methods only calculate edge statistics per region without using relative density variation between local patch and neighborhood (which is an important cue to differentiate the fine-scale fracture patterns under acquisition conditions variability).

Inspired by these shortcomings, we propose the Adaptive Block-wise Relative Edge Density (ABRED) pipeline, a physically informed and interpretable feature design that systematically addresses each deficiency. For (i), quadtree decomposition is steered by local gradient variance. High structural complexity regions (e.g., compact fracture networks) force recursion, while homogenous regions are only coarsely partitioned—a guarantee that intra-block edge distributions are smooth with respect to statistical moments. For (ii), relative edge density is defined as the normalized ratio of a block’s edge count and the mean edge count across its eight neighbor blocks, which effectively mitigates multiplicative illumination bias in our experiments without requiring explicit photometric preprocessing. For (iii), we incorporate multi-scale representation through Gaussian blurred edge density maps at three progressively smoothed resolutions that account for scale discrepancy caused by variable camera–rock distances. Most importantly, ABRED endows geological priors directly into the computation pipeline, achieving high classification accuracy, low inference latency on CPU, and full interpretability and making it a trustworthy and resource-aware candidate for periodic tunnel safety monitoring, with potential for future optimization on embedded platforms.

## 2. Review of Surrounding Rock Image Classification Methods

### 2.1. Current Status of Tunnel Surrounding Rock Fracture Image Classification

Image-based crack classification of fracture states mostly learns to infer the fragmentation severity in rock mass through quantification of geometrical features, e.g., joint plane density, aperture width, or surface roughness [15]. Historically, such analyses relied on manually intensive tracing of fractures, followed by calculation of, e.g., the fracture area ratio or the average spacing. More recently, generic computer vision tools, e.g., from traditional texture descriptors (LBP, HOG, Gabor) to deep learning models and architectures, have been used for automatizing this task. However, as conventional features, they are inherently limited by fixed spatial partitioning and lack domain-semantic design principles. They neither reflect the high variability in spatial distribution of fracture patterns, nor utilize illumination-robust normalization. In the field environment, the resulting features are then brittle against real-world imaging conditions [16].

Recent years have seen several advancements for deep-learning based methods for tunnel geotechnical analysis. Notable examples include a convolutional architecture specifically designed to identify core fractures and infer their incidence angles [17]; a semi-supervised learning scheme specifically developed for rock mass type classification, with the goal to supporting real-time TBM decision making [18]; a hybrid segmentation approach coupling U-Net and clique-aware CNN to perform high-resolution crack delineation on tunnel linings [19]; an attention-enhanced U-Net architecture involving Convolutional Block Attention Modules (CBAM) to enhance the accuracy of fracture localization on the face of the excavated tunnel [20]; a CNN-based approach to directly learn the recognition of structural discontinuities using high-resolution, close-range photographs of these [21]; data augmentation techniques in the form of generative noise models to address limited availability of annotated training samples [22]; T Rai C developed as an end-to-end AI platform to allow for real-time 360° panoramic mapping of the tunnel face and discontinuity characterization using computer-automated means [23]; a multimodal fusion between TBM working telemetry and vision-based geological interpretation leveraging a hybrid unsupervised–supervised training scheme [24]; a critical review of the core image-based imaging techniques used in vein and fracture quantification [25]; and Oil-Net, which is a vision-based diagnostic system developed for global assessment of underground working places [26].

Nonetheless, none of these approaches dynamically adjust region granularity to match the multi-scale nature of fracture patterns nor do they inherently compensate for lighting inconsistencies. The uniform tiling strategy underlying LBP, HOG, and Gabor, along with global or fixed-block edge counting schemes used in absolute density methods, overlooks the intrinsic spatial heterogeneity of fracture occurrence, making them ill-suited for accurate, robust, and field-deployable tunnel rock assessment.

### 2.2. Adaptive Blocking and Edge Density Features

Further, standard absolute edge density approaches calculate edge counts either at a global level or within fixed and pre-computed blocks, lacking local context contrast. Importantly, the relative density between a local window and its spatial neighbors, which is a strong and informative discriminative cue on geologically inhomogeneous scenes, is always discarded by contemporary fixed-partition frameworks.

Intuitively, mainstream handcrafted features such as LBP, HOG, and Gabor as well as fixed-grid edge density solutions suffer from three mutually interwoven difficulties when applied to tunnel surrounding rock images: (i) Spatial scale heterogeneity: Fracture scales range from fine (micro-cracks) to large (persisted joints) orders of magnitude, prohibiting uniform partitioning from serving. (ii) Illumination instability: multiplicative lighting variations (spotlighting, shadow shading), which disproportional distort absolute edge response, invalidate feature regularity. (iii) Single scale: A single resolution can hardly capture simultaneously small (localized) fractures and large-scale discontinuities.

**Figure 1:**
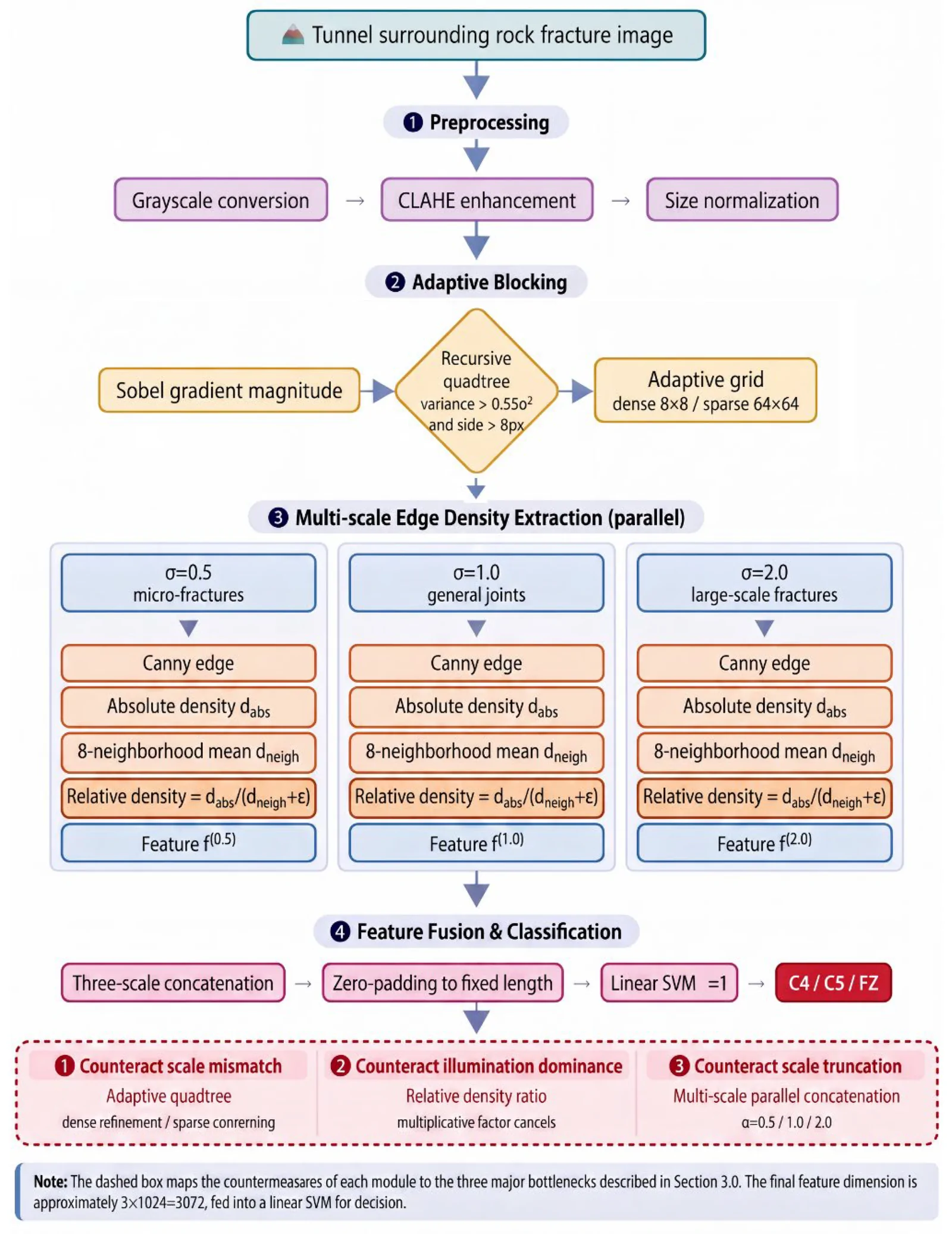
Technical roadmap of ABRED.

To holistically address these limitations, Figure 1 illustrates the unified technical architecture of Adaptive Block Relative Edge Density (ABRED), delineating each challenge to a dedicated, physically motivated module. Spatial non-uniformity is addressed with gradient variance-guided quadtree decomposition, recursively splitting off high-complexity areas with fine partitions while maintaining coarse granularity in homogeneous parts of the image. Illumination sensitivity is combated by computing relative edge density as the ratio of a block’s edge count to the average of its eight neighbors to automatically cancel multiplicative gain effects. Multi-scale variability is represented by parallel extraction of Gaussian smoothed edge density descriptors at the three levels σ = 0.5, 1.0, 2.0, which are then catenated into a combined multi-resolution representation. This schematic form the conceptual backbone to Section 3. Section 3.1, Section 3.2, Section 3.3, Section 3.4 and Section 3.5 will provide formal mathematical formulations, rationales for parameter selection, and step-wise computational procedures for each component.

## 3. Methodology

### 3.1. Design Motivation: From the Essence of the Classification Task

Before introducing algorithmic details, it is first necessary to motivate the geomechanically based classification reasoning for every component of ABRED. The three-class classification of tunnel surrounding rock—low, moderate, and high fracture density—is not a standard textural classification problem. Its inherent challenges arise from the fact that discriminative ability lies not in binary edge detection but rather in the spatial variability of edge density relative to its neighborhood. Using generic texture descriptors without adapting to their application domain leaves three failure modes:

(1) Spatial scale mismatch: Uniform partition schemes overlook the inherent sub-metric heterogeneity of fractures. In relatively intact rock domains, overly fine-grained sampling provides numerous low-magnitude, statistically unstable edge responses, injecting spurious noise that dilutes classifier confidence for the “low-density” class. In the meantime, highly fractured regions are partitioned into too coarse blocks that collapse multiple discontinuities into a single statistical unit, homogenizing localized density highs and flattening diagnostically critical structural gradients.
(2) Illumination-induced bias: Features extracted from raw edge counts are intrinsically contrast dependent. Identical geology imaged with different illumination geometries (e.g., a different lamp angle or intensity) can lead to intra-class absolute density differences of orders of magnitude larger than inter-class differences among distinct fracture states. Classifiers may therefore learn to treat artifacts of illumination more strongly than geo-mechanical signatures, attracting decision surfaces driven by illumination geometry rather than fracture severity.
(3) Inadequate resolution: In situ tunnel faces contain fractures that cover an extensive morphological range from sub-pixel scale micro-cracks to macro-scale pervasive joints reaching tens of pixels in length. A statically chosen resolution can at best sacrifice either fidelity or robustness. Higher resolutions resolve micro-fractures while exaggerating dust, moisture, and sensor artefacts; lower resolutions smoothen noise while throwing out structure-preserving small-scale patterns.

Accordingly, ABRED’s modular architecture is deliberately structured as a targeted response to each limitation:

(1) Adaptive blocking adjusts mismatches in the spatial scale by intelligently adjusting block dimensions based on local gradient variability, ensuring consistent and robust edge distribution properties across different geologies;
(2) Relative density normalization mitigates illumination-induced distortions by scaling each block’s edge count relative to the average edge count of its eight adjacent blocks, effectively converting lighting-dependent intensity values into robust, illumination-invariant contrast descriptors;
(3) Multi-scale feature fusion addresses these drawbacks by fusing edge density maps with each smoothed using Gaussian kernels at three distinct yet complementary scales (σ = 0.5, 1.0, and 2.0) to jointly describe both subtle textural boundaries and broad structural patterns.

Steps for the realization of image preprocessing, adaptive quadtree partitioning, gradient-based edge localization, neighborhood-relative density computation, and multi-resolution feature aggregation are explained in Section 3.2, Section 3.3, Section 3.4, Section 3.5 and Section 3.6. All parameter choices, such as variance thresholds, smoothing kernels, and neighborhood definitions, are properly optimized based on the main goal of maximizing the inter-class discrimination while minimizing intra-class jittering.

### 3.2. Image Preprocessing

Raw color images at tunnel faces typically have low visual contrast as a result of non-uniform illumination, airborne dust and water films, and sensor constraints. A domain-aware preprocessing pipeline is adopted to prepare the inputs to extract robust features.

Luminance is converted through the ITU-R BT.601 standard [27] (Y = 0.299R + 0.587G + 0.114B) to discard color data with a resulting grayscale encoding that retains structural detail and reduces computational overhead and avoids all chromatic noise not related to the underlying fracture characteristics.

Second, Contrast Limited Adaptive Histogram Equalization (CLAHE) is used with a tile size of 16 × 16 and a clip limit of 3.0, balancing between local contrast adjustment and noise filtering. This is especially important where the signal is weak, e.g., over shadowed or dusty rock faces.

**Figure 2:**
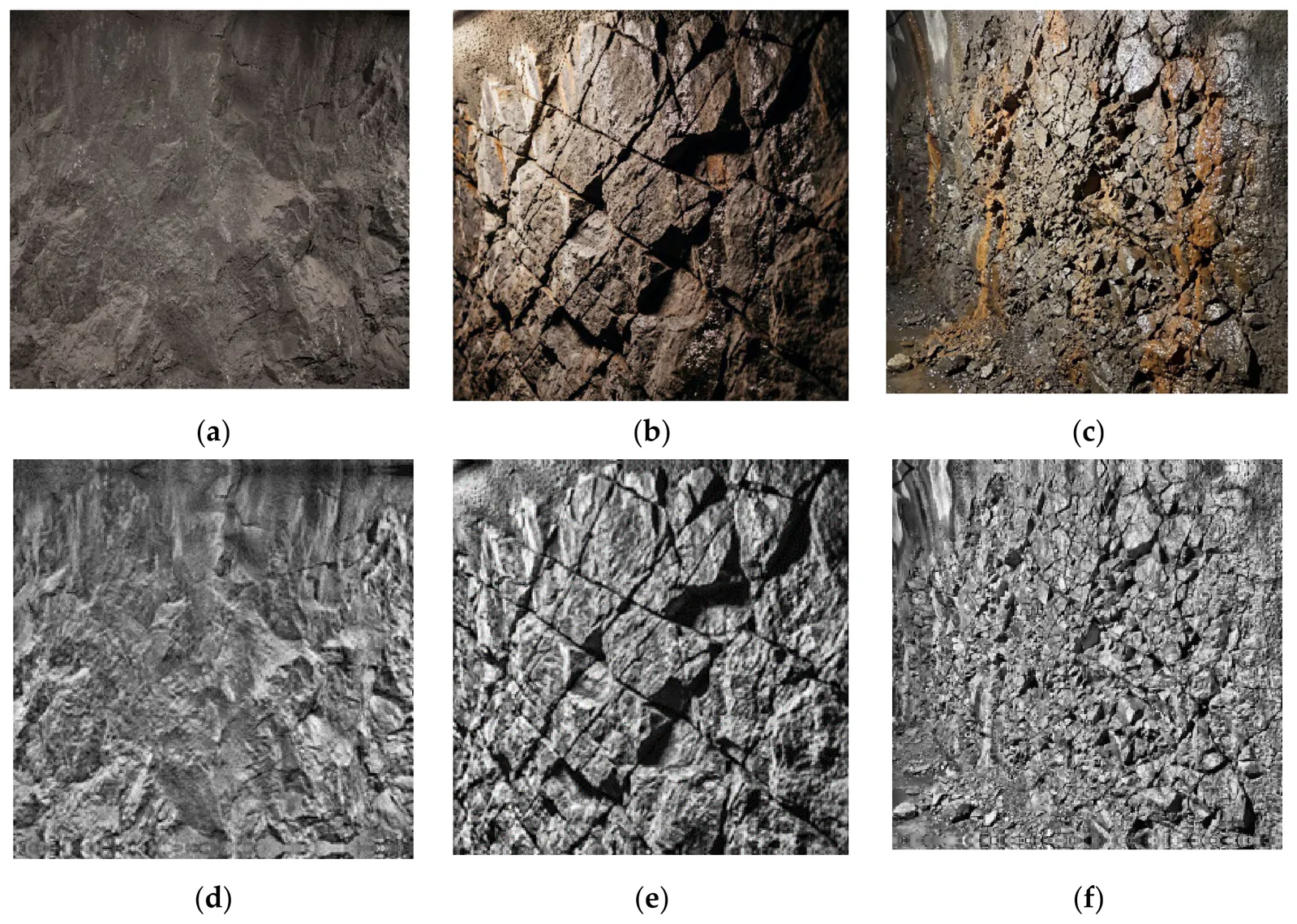
Schematic diagram of image preprocessing and grayscale equalization for three fracture states. (**a**) Original site image with sparse fractures. (**b**) Original site image with dense fractures. (**c**) Original site image with extremely dense fractures. (**d**) Grayscale equalization result for sparse fractures. (**e**) Grayscale equalization result for dense fractures. (**f**) Grayscale equalization result for extremely dense fractures.

Third, all images are resampled to a fixed dimension of 256 × 256 pixels while preserving original aspect ratio and padding the shorter dimension (i.e., with mirror (reflection) maintain geometric integrity, without inducing spurious edge discontinuities at edges. Figure 2 illustrates the preprocessing outcomes—including grayscale conversion, CLAHE enhancement, and resizing—for representative images from the three fracture classes.

### 3.3. Adaptive Blocking Based on Gradient Variance

Conventional uniform partitioning strategies, such as fixed 16 × 16 or 32 × 32 pixel grids, fail to account for the spatially heterogeneous nature of fracture distribution. This results in excessive, statistically redundant subdivisions over intact rock zones, while simultaneously over smoothing and obscuring fine-scale structural details in highly fractured regions. To overcome this limitation, we adopt a data-driven, recursive quadtree decomposition guided by local structural complexity that is quantified via the variance of gradient magnitude within each candidate block. Gradient magnitude is computed using the Sobel operator, which provides robust first-order edge strength estimation while maintaining computational efficiency and noise resilience.

(1) $$M(x,y)=\sqrt{G_{x}^{2}+G_{y}^{2}},$$

Taking the entire image as the initial block, let the variance of gradient magnitudes within the current block *B* be $\;\sigma_{B}^{2}\;$ and the minimum block size be $\sigma_{min}$. If $\sigma_{B}^{2}$ > T (threshold) and the block side length is greater than $\sigma_{min}$, the block is divided into four equal parts recursively; otherwise, the division stops. The threshold is set as $T=\alpha \times \sigma_{global}^{2}$, with α = 0.55 determined using grid search.

**Figure 3:**
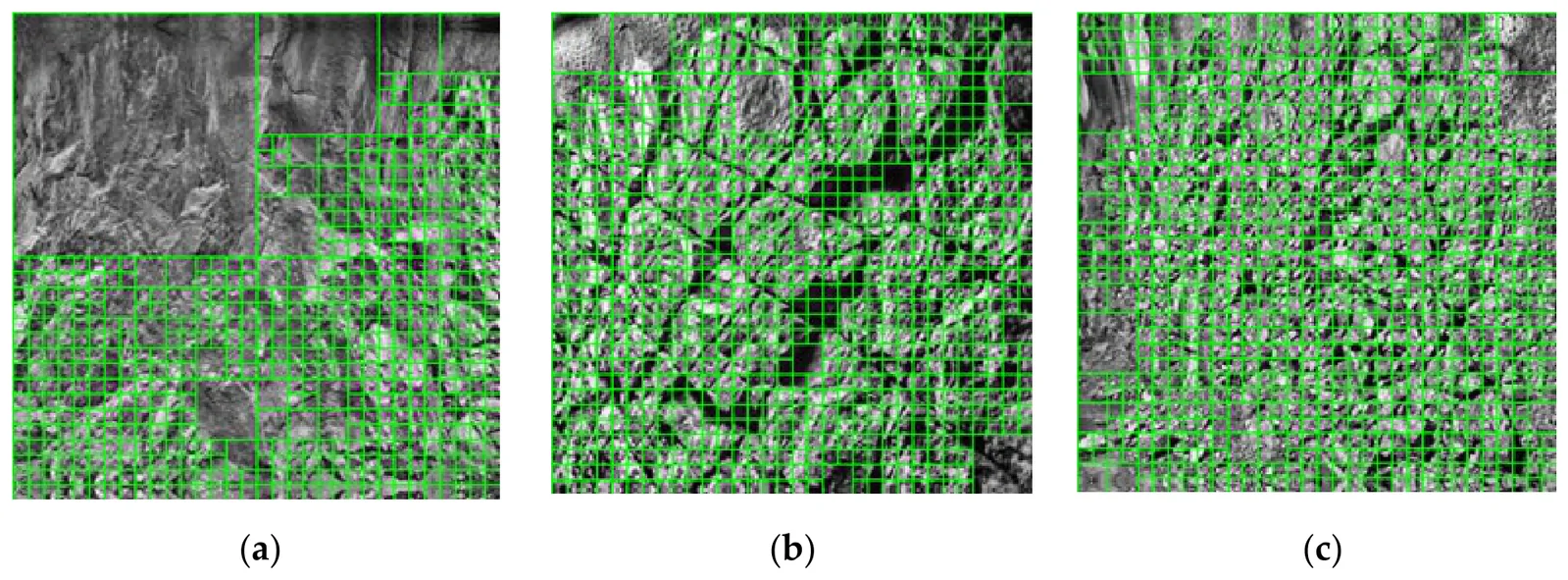
Adaptive quadtree segmentation results overlaid on gradient magnitude images for three fracture classes. (**a**) Segmentation for sparse fractures. (**b**) Segmentation for dense fractures. (**c**) Segmentation for extremely dense fractures.

We adaptively tailor block granularity by the local structural complexity. In areas of high fracture density—and hence high gradient magnitude variance—recursive partitioning aggressively subdivides blocks down to 8 × 8 or 16 × 16 pixels and encodes fine-scale discontinuity information. However, in smooth, undamaged rock zones, with low variance, partitioning stops early, leaving behind coarse blocks of 32 × 32 or even 64 × 64 pixels. This avoids over-smoothing while maintaining statistical fidelity. The resulting hierarchal segmentation thus produces a spatially adaptive and resolution-efficient partitioning. Figure 3 provides an illustration of this phenomenon by overlaying the quadtree split bounds on the image and a superimposed synthetic gradient map. As can be observed, the high variance area of the middle, which corresponds to a simulated dense fracture cluster, is composed of small, closely packed blocks (16 × 16 and 8 × 8), while the surrounding homogeneous background is covered by significantly larger blocks (64 × 64), providing evidence that the method effectively optimizes partitioning granularity according to local structural saliency.

### 3.4. Edge Detection and Absolute Edge Density

Before computing the absolute edge density, a binary edge map must be obtained. After adaptive blocking, the Canny operator is used to extract the binary edge map. The Canny thresholds are adaptively set based on the global gradient mean $\mu_{M}$: low threshold $t_{low}=0.4\times \mu_{M}$, high threshold $t_{high}=0.8\times \mu_{M}$; Gaussian smoothing parameter $\sigma_{Canny}$ = 1.0 pixel, which suppresses dust noise while preserving real fractures. Given the resulting binary edge map E, where E(x,y)=1 for edges, the absolute edge density for each block is defined as follows:

(2) $$d_{i}^{abs}=\frac{1}{\left|B_{i}\right|}\sum_{\left(x,\right.\left.y\right)\in B_{i}}E(x,y)$$

**Figure 4:**
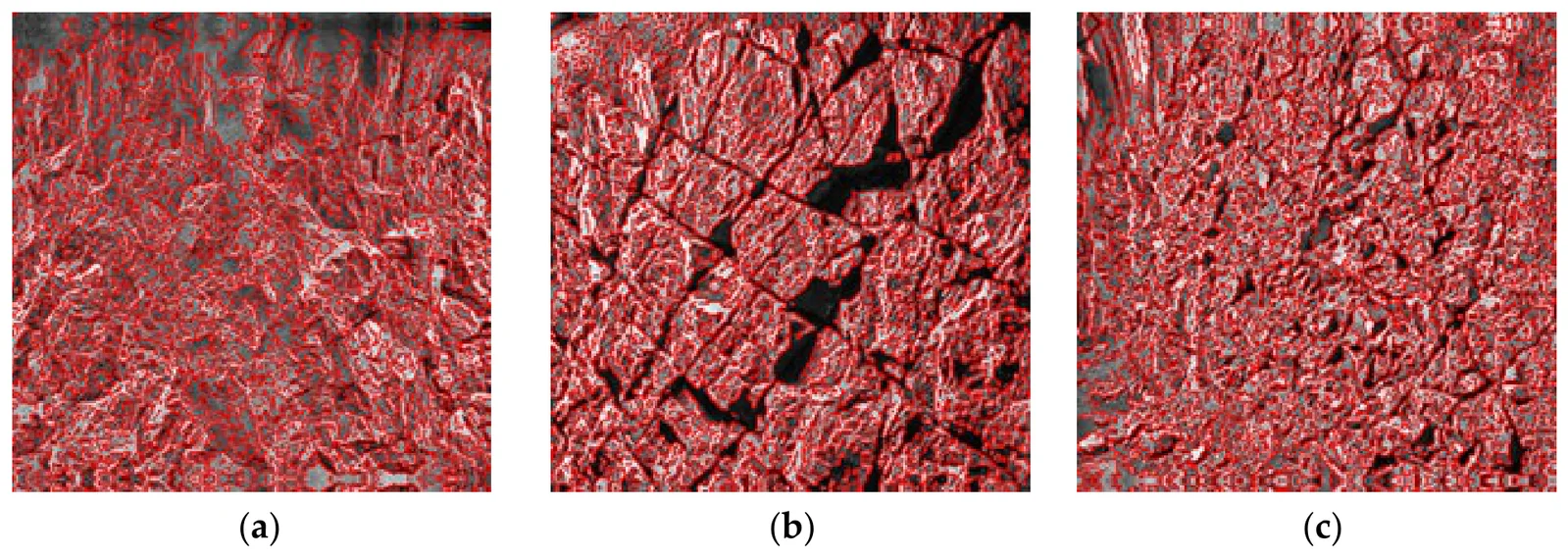
Canny edge detection results for three typical images. (**a**) Sparse fractures. (**b**) Dense fractures. (**c**) Extremely dense fractures.

This reflects the degree of fracture density within the block. Intact rocks typically yield $d_{i}^{abs}<0.05$, while large joints may exceed 0.3. However, absolute density is sensitive to global illumination variations, necessitating the introduction of relative density. Figure 4 presents the binary edge maps obtained by the Canny operator for the same three exemplar images, demonstrating the extraction of edge structures at different fracture densities.

### 3.5. Relative Edge Density

To overcome the sensitivity of absolute density to multiplicative global illumination changes, we introduce the relative edge density. For each block $B_{i}$, we consider its eight-neighborhood set N(i) (using only existing neighbors at boundaries) and compute the neighborhood average absolute density:

(3) $${\bar{d}}_{neigh}\left(i\right)=\frac{1}{\left|N_{i}\right|}\sum_{j\in N_{i}}d_{j}^{abs}$$

The relative edge density is then defined as follows:

(4) $$r_{i}=\frac{d_{i}^{abs}}{{\bar{d}}_{neigh}\left(i\right)+\epsilon }$$

where ϵ = 0.005 is a smoothing factor to prevent division by zero. If a block’s density is significantly higher than its neighborhood, $r_{i}\gg 1$. If lower, $r_{i}<1$. In uniform areas, $r_{i}$ ≈ 1. The multiplicative illumination factor c cancels out in the ratio, ensuring inherent robustness at the edge count level. However, we emphasize that exact cancellation of multiplicative illumination factors is not guaranteed after CLAHE preprocessing, adaptive Canny thresholding, and binarization; therefore, ABRED should be characterized as empirically robust to the tested perturbations rather than theoretically illumination invariant.

Nevertheless, we note that this cancellation is only approximate under idealized assumptions. Exact multiplicative invariance does not strictly hold after CLAHE preprocessing, adaptive Canny thresholding, and binarization are applied. Hence, ABRED is properly described as empirically robust to illumination variations under the tested conditions, rather than theoretically invariant. Quantitative evidence of this robustness is provided in Section 5.3.

**Figure 5:**
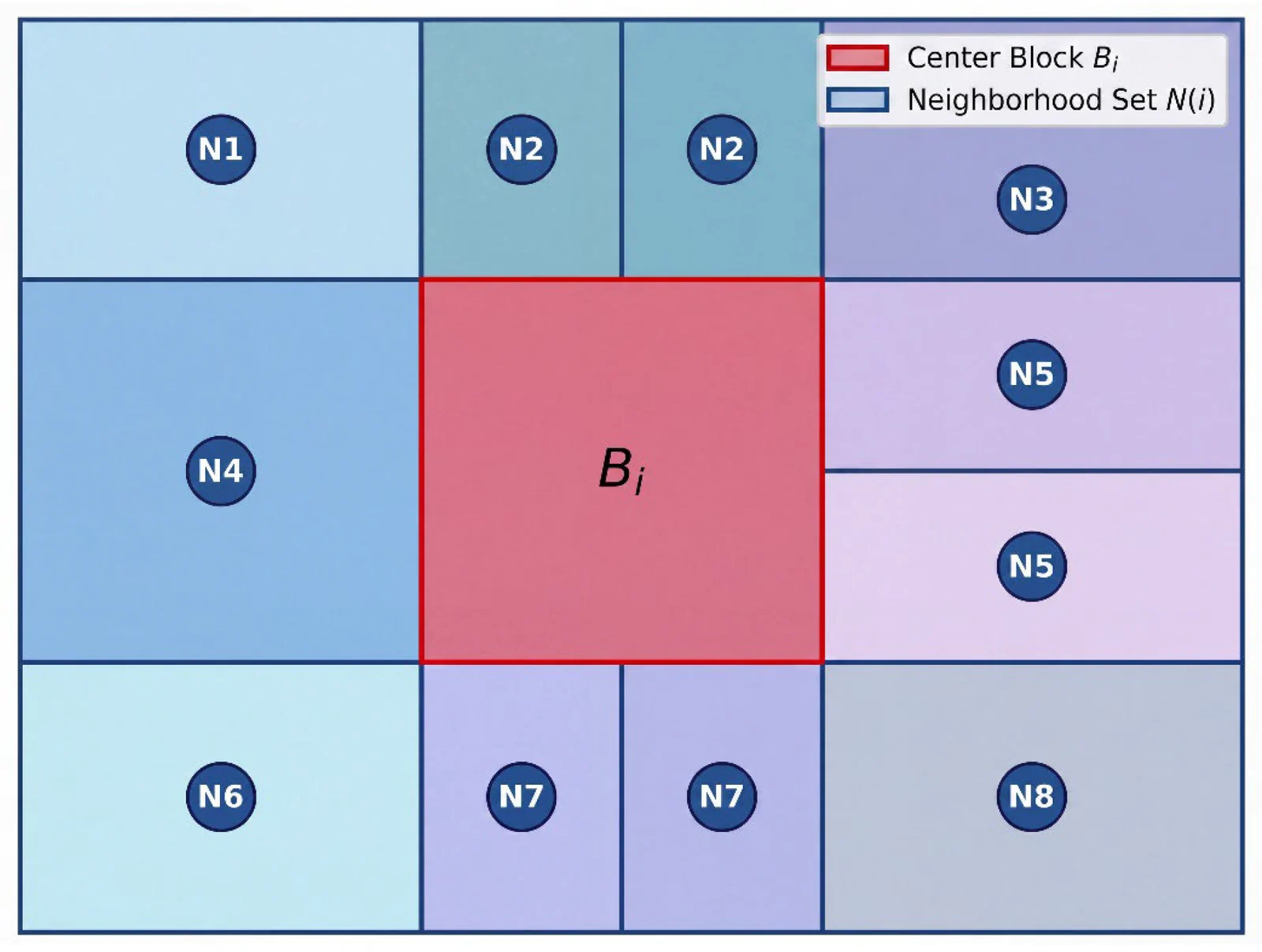
Schematic diagram of eight-neighbor identification for unequal quadtree blocks. The center block Bi (red) is adjacent to blocks of varying sizes. All blue blocks sharing any portion of a boundary with Bi, including cases where one side corresponds to multiple smaller blocks (e.g., *N*2 and *N*5), are included in the neighborhood set *N*(i).

For a given block Bi, its eight-neighborhood set *N*(i) is defined as the set of all leaf blocks that share any portion of a boundary with *B*i (including partial boundaries). In a quadtree decomposition, a large block may be adjacent to multiple smaller blocks along a single side (e.g., a 32 × 32 block adjacent to four 16 × 16 blocks on its right). In such cases, all of these smaller blocks are included in the neighborhood set. The same principle applies when a smaller block is adjacent to a larger block, where the larger block is included as a single neighbor. At image boundaries, only existing neighbors are used; no padding or extrapolation is applied. This ensures that the neighborhood average ${\bar{d}}_{neigh}\left(i\right)$ accurately reflects the local edge density context, regardless of block size variations. Figure 5 provides a schematic illustration of this neighbor identification procedure for a representative quadtree configuration with unequal block sizes.

All $r_{i}$ are arranged in a fixed raster scan order (top to bottom, left to right) over the leaf blocks of the quadtree to form a feature vector $f\in R^{K}$. Each leaf block is uniquely identified by its spatial position (coordinates of its top-left corner) and its size, ensuring that the ordering is deterministic and reproducible. It is important to note that the same dimension index in the feature vector does not necessarily correspond to the exact same physical location across images with different quadtree structures because the number and arrangement of leaf blocks vary. However, the raster scan order ensures that each dimension index consistently corresponds to a block with a similar relative spatial position (e.g., a given index may correspond to the top-left region of the image) across all samples. The linear SVM treats the entire feature vector holistically and learns discriminant weights for each dimension based on statistical co-occurrence across the training set; explicit pixel-wise correspondence is not required for the classifier to be effective, as demonstrated by the strong classification performance reported in Section 5. This approach is standard in variable size feature extraction methods that employ zero-padding to achieve fixed dimensionality.

It is important to clarify that the fixed dimension after zero-padding does not imply pixel-wise spatial correspondence across images with different quadtree structures. Instead, each dimension index in the raster scan order corresponds to a block with a statistically similar relative spatial position (e.g., the top-left region, the upper-middle region) across the dataset. The linear SVM learns discriminant weights for each dimension based on the statistical co-occurrence of edge density patterns across the training set, rather than relying on exact pixel-level geometric alignment. To empirically validate that the classification performance does not trivially arise from the total number of blocks K (which could be encoded by the zero-padded tail), we conducted a control experiment where only the scalar value K was used as the sole feature. This K-only baseline achieved only 64.5% accuracy, substantially lower than the 87.6% accuracy of the full ABRED feature (see Section 5.7). This confirms that the discriminative power of ABRED is primarily driven by the relative density distribution within the blocks, not by the block count or the padded tail.

**Figure 6:**
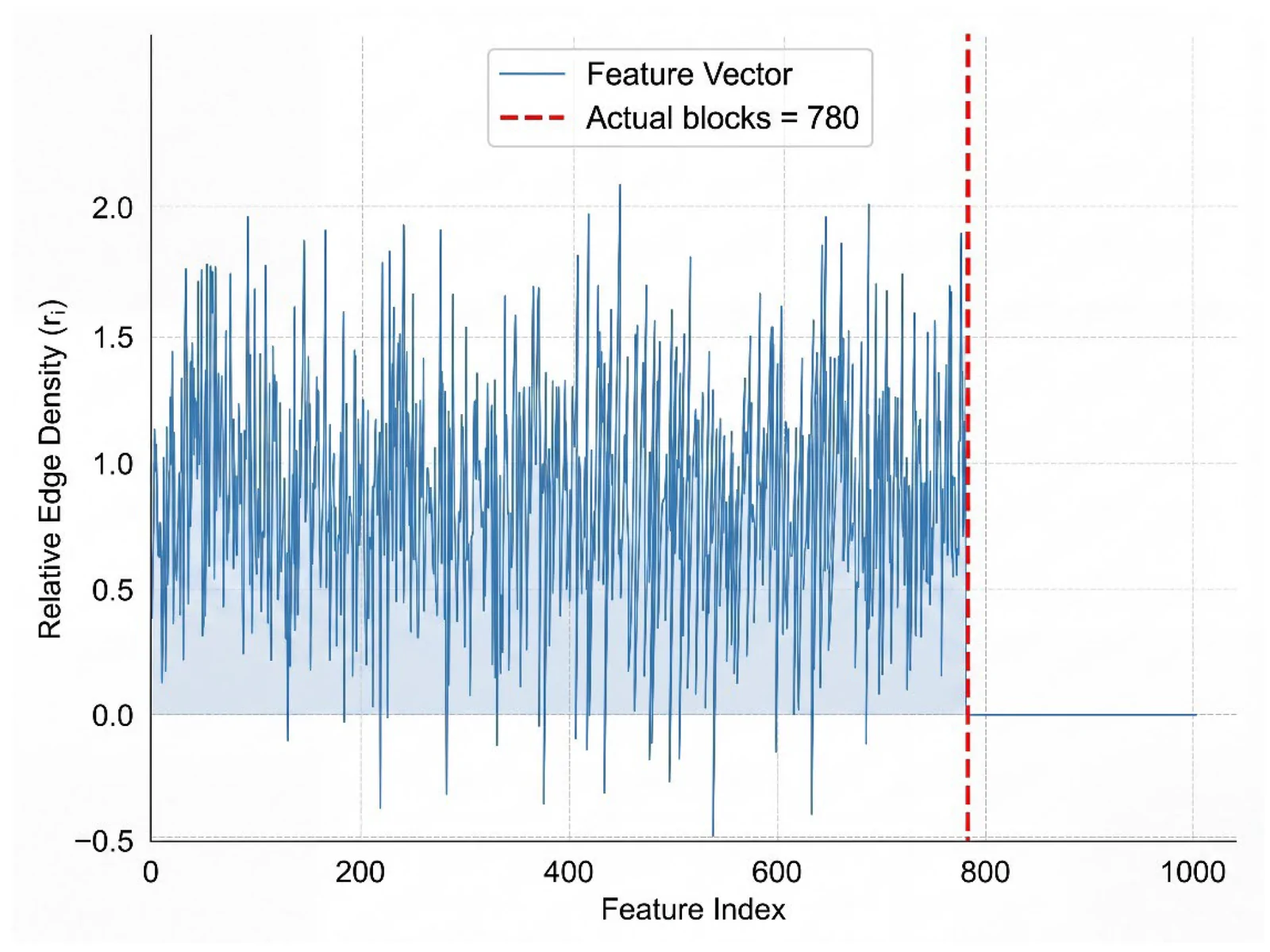
Schematic diagram of feature vector tail zero-padding.

**Figure 7:**
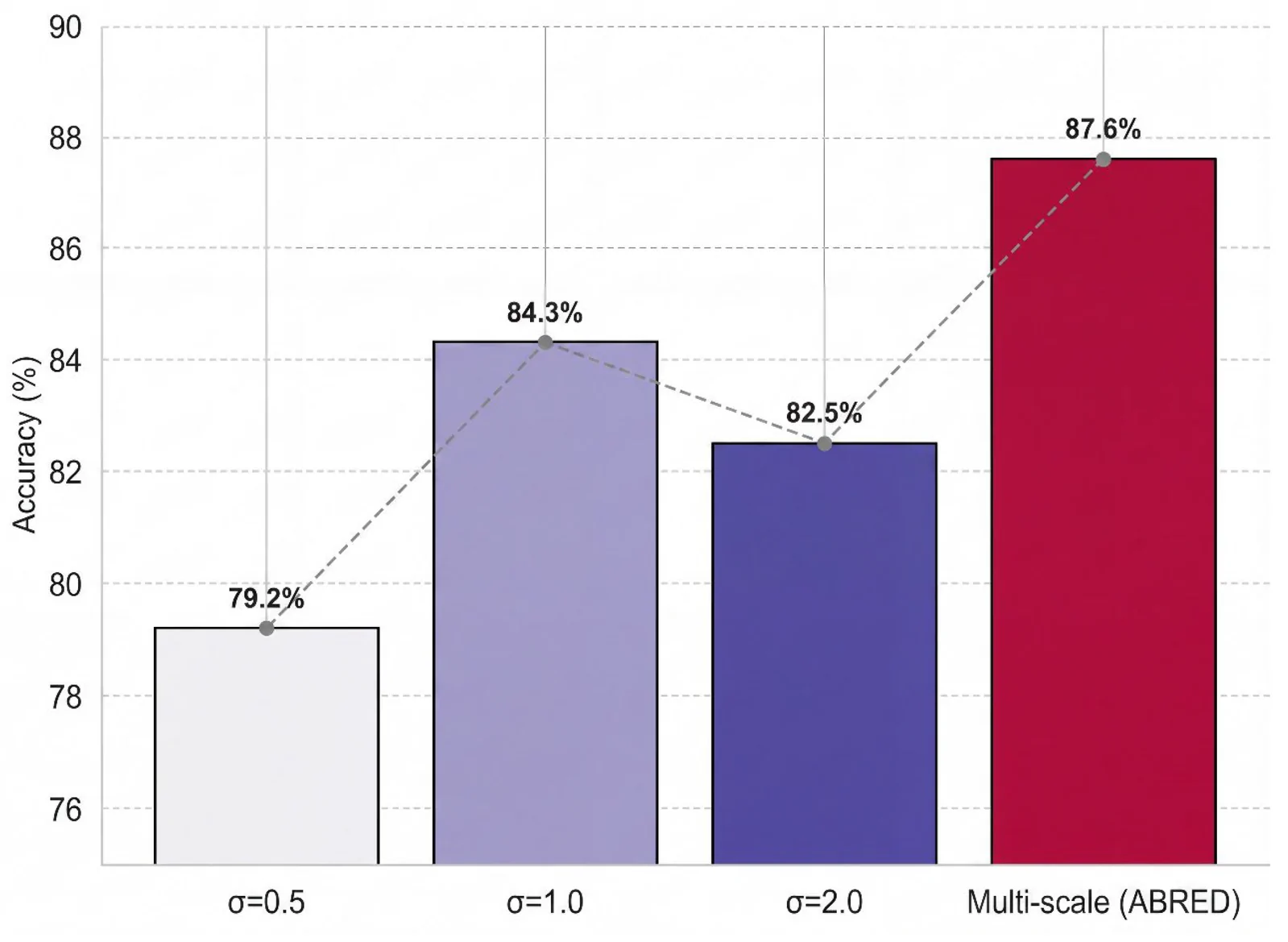
Bar chart of multi-scale expansion effect.

Since the number of blocks K varies per image (approximately 200 to 900), we set a maximum length $K_{max}=1024$ and zero-pad the tail of shorter vectors to ensure a fixed dimensionality. Figure 6 (newly added schematic) illustrates this neighbor identification procedure for a representative quadtree configuration. It is important to note that zero-padded entries are simply zeros and are not masked is applied during linear SVM training or inference; the full 1024-dimensional vector, including the zero-padded positions, is used directly. Because the zero-padded positions are constant across samples with similar K and the linear SVM treats these as features with a fixed zero value for those samples, these positions do not carry class discriminative information beyond a constant bias contribution. Figure 7 visualizes this mechanism. For a block count of 780, only the first 780 entries in the feature vector hold meaningful relative density measurements; all remaining positions are padded with zeros, ensuring no extraneous or misleading data is introduced.

### 3.6. Multi-Scale Extension

For greater resolution robustness, multi-scale Gaussian smoothing is proposed. Three scales are adopted: $\sigma_{s}\in \{0.5,1.0,2.0\}$. The result with $\sigma_{s}$ = 0.5 filters out fine-scale micro-fractures. The result with $\sigma_{s}$ = 1.0 captures general joint information. The result with $\sigma_{s}$ = 2.0 preserves large-scale fractures while suppressing noise. For each scale, the whole adaptive blocking, edge finding, and relative density computation is done and gets feature vectors $f^{\left(0.5\right)}$, $f^{\left(1\right)},f^{\left(2\right)}$. These three vectors are concatenated to form the final multi-scale feature: $f_{final}=[f^{\left(0.5\right)}$; $f^{\left(1\right)}$; $f^{\left(2\right)}]$. Since each single-scale vector has a maximum length of 1024, the final feature dimension is approximately 3072.

For the three-class fracture problem treated here (i.e., classifying the surrounding rock of a tunnel into low, moderate, and high fracture density), a linear SVM is computationally efficient and highly discriminative when trained in the proposed multi-scale ABRED feature space. Compared with single-scale representations, such a multi-resolution formulation provides significantly greater discriminative power. Empirically evaluated, combining σ = 0.5, 1.0, and 2.0 scales boosts overall classification performance (which would have been 84.3% for the best-performing single scale σ = 1.0) from 84.3% to 87.6%, demonstrating the utility of scale fusion. As shown in Figure 6, multi-scale ABRED outperforms each single scale, demonstrating resilience to structural variations and supporting its practical use in real-world tunnel assessment tasks.

### 3.7. Complexity Analysis

In practical tunnel engineering, where decisions must often be made through quick analysis deploy, ability is a key constraint of computational efficiency. We robustly benchmark ABRED runtime on commodity hardware. To run on a 256 × 256 grayscale input with n = 65,536 pixels, Sobel-based gradient magnitude computation has O(n) complexity and takes 0.02 s. Adaptive quadtree partitioning has theoretically bounded O(n log n) complexity, benefiting from early termination in homogeneous regions, resulting in near-linear empirical behavior and 0.05 s mean runtime in Python. Canny edge detection, including Gaussian smoothing, gradient estimation, non-maximum suppression, and double-threshold hysteresis, also has O(n) complexity and required 0.06 s. Absolute and relative density computations loop only over the remaining final set of blocks K ≈ 200–900; O(K) overhead is thus ignorable.

**Figure 8:**
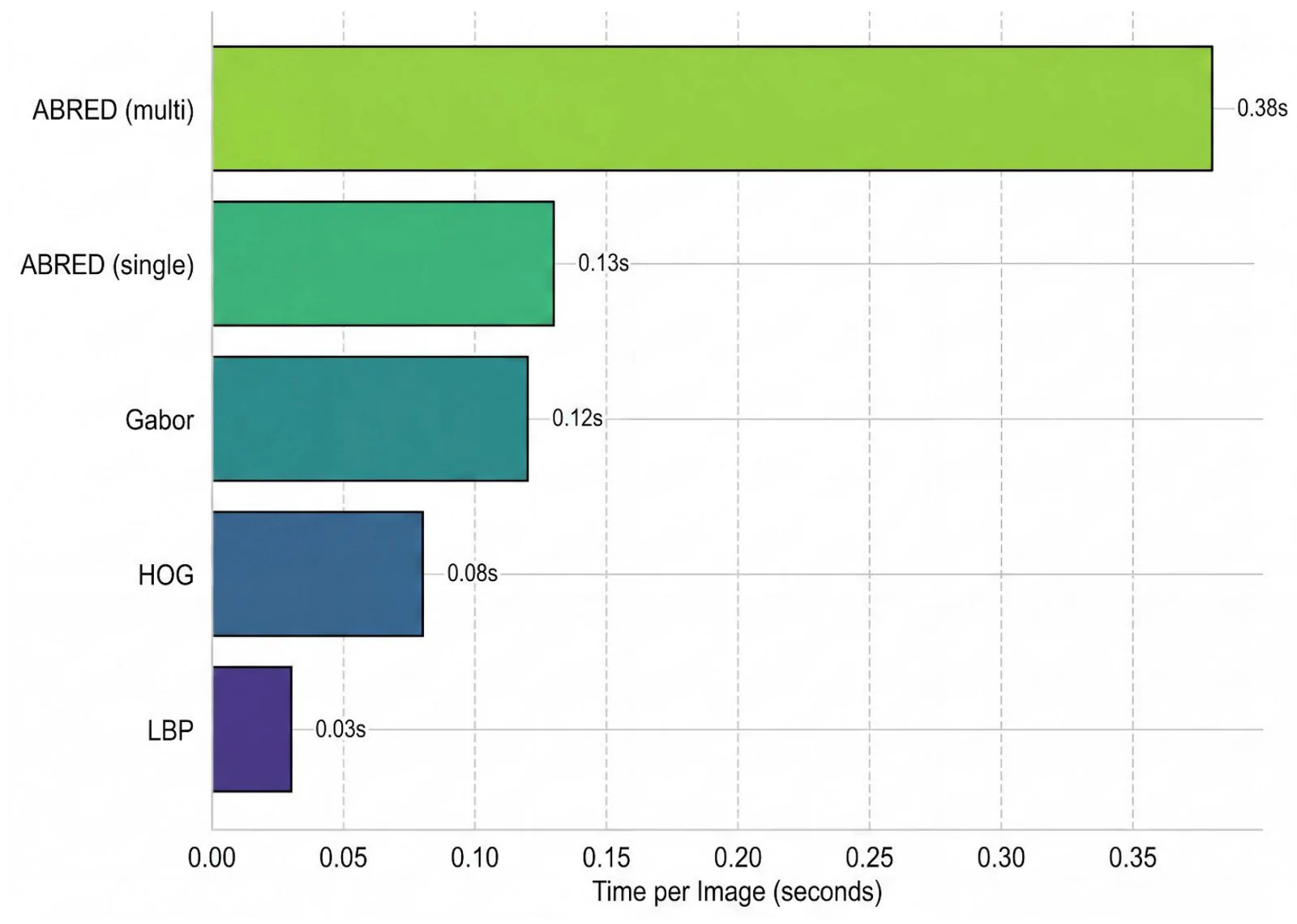
Horizontal bar chart of computation time comparison across different feature extraction methods on a single core of an Intel i5-1135G7 CPU. Methods include ABRED (single scale and multi-scale), LBP, HOG, Gabor, FBD (Fixed-Block Absolute Density), FBR (Fixed-Block Relative Density), and ABD (Adaptive-Block Absolute Density). The bars represent the average processing time (in seconds) per 256 × 256 grayscale image, demonstrating that ABRED, despite being slower than the simplest descriptors, still achieves throughput (2.6 fps for multi-scale) suitable for periodic tunnel face assessment.

On a single core of an Intel i5-1135G7 CPU, the single-scale ABRED pipeline takes about 0.13 s for a single image. Scaling to three scales runs at 0.38 s per image, corresponding to about 2.6 frames per second. While slower than lightweight descriptors such as LBP (0.03 s/image), this throughput easily allows for periodic tunnel face scanning (e.g., every 2–3 s during TBM advance or manual inspection), and safely stays within reasonable bounds for safety critical assessment routines using standard CPU hardware. Further acceleration through GPU offloading, optimized C++ kernels, or model quantization could enable genuine real-time operation on embedded platforms, although we have not validated such deployment in this study. Importantly, this modest computational burden is more than justified by ABRED’s significant improvement in classification accuracy, as well as its physical interpretability, making it both technically and operationally compelling for CPU-based periodic assessment Figure 8 provides a horizontal bar chart comparing the per-image computation time of ABRED (both single scale and multi-scale) with that of the baseline handcrafted features (LBP, HOG, and Gabor), illustrating that ABRED’s runtime remains within practical bounds for periodic field deployment while delivering superior classification accuracy.

## 4. Experimental Design

### 4.1. Dataset

In lieu of a publicly available and specifically domain-based benchmark on tunnel surrounding rock imagery, we built a custom-made, expert-annotated dataset with 1200 high-resolution imagery acquired in a variety of positions, including both the face and sidewalls of the tunnel, inside the Fengyanshan Tunnel. All images were uniformly resampled to 256 × 256 pixels so as to have input homogeneity and preserve structural integrity. The ground truth labels were assigned by authorized geological engineers based on the classification of surrounding rock quality from China’s national standard, the Highway Tunnel Design Code (JTG D70-2014) [28]. The annotations incorporate field observations and detailed lithology and structural descriptions, as well as post-construction monitoring records to be geo-mechanically accurate and applicable. The final class distribution, namely, low, moderate, and high fracture density, is listed in Table 1.

To prevent data leakage and ensure a realistic evaluation of field performance, the 70/30 train-test split was performed at the acquisition location level rather than the image level. Specifically, all images captured from the same tunnel face or sidewall section were grouped and assigned exclusively to either the training set or the test set. This grouped split strategy ensures that the test set consists of entirely unseen tunnel sections, providing a more rigorous assessment of the model’s generalization capability.

The ground truth labels (C4, C5, and FZ) were independently assigned by three senior geological engineers, each with over 10 years of experience in tunnel engineering and rock mass classification. The labeling was guided by China’s national standard, the Highway Tunnel Design Code (JTG D70-2014), supplemented by field observations and post-construction monitoring records. Disagreements among the experts were resolved by a majority vote, ensuring the generation of a high-quality, consensus-based label set.

The dataset was partitioned using a stratified grouped split based on acquisition location. Specifically, 70% of the images were allocated to the training set, and the remaining 30% were reserved as the held-out test set. The test set was strictly isolated from the outset and was not used in any form during model development or parameter tuning. For hyperparameter selection, a 5-fold cross-validation was performed internally on the training set to optimize the key parameters, including the quadtree variance threshold coefficient α, the Canny edge detector thresholds, and the SVM penalty parameter C. The validation fold predictions guided the selection of the optimal parameter configuration (α = 0.55, C = 1, etc.). Only after all parameters were fixed was the final model evaluated on the unseen test set to obtain the unbiased performance metrics reported in this paper. This strict separation ensures that the test set results reflect genuine generalization performance on previously unseen tunnel faces.

The dataset was partitioned by class: 70% of all images per class for training and the remaining 30% for independent testing, ensuring balanced and thus statistically valid testing. All images were manually curated before partitioning, removing those with extreme degradation, such as total defocusing, extreme overexposure (e.g., saturated highlights that occlude texture), or significant occlusion (e.g., by personnel, equipment, or debris), ensuring quality analysis ready inputs.

Image acquisition and preprocessing followed best practices found in recent surveys of prevailing, vision-based, structural-defect detection practice [29]. Capture was undertaken via calibrated, handheld DSLR and industrial-grade cameras with controlled ambient lighting. This is consistent with currently recommended standards in the industry for tunnel face documentation. While emerging robotic platforms and autonomous excavation systems—for example, recently surveyed—display exciting promise for future automated, continuous imaging, manual acquisition remains the operational de facto standard for geological characterization in working, active tunnel conditions (due to versatility, adaptability to confined areas, and real-time expert control). All 1200 images from diverse perspectives on tunnel faces and sidewalls of Fengyanshan Tunnel were resampled uniformly to 256 × 256 pixel format to standardize input dimensions without sacrificing diagnostic spatial information.

### 4.2. Comparison Methods

To rigorously assess the discriminative capability of the proposed ABRED feature for tunnel surrounding rock classification, we conduct a systematic comparative evaluation against six well-established baseline descriptors, with each representing a distinct design philosophy in texture and structural analysis.

Fixed-Block Absolute Density (FBD): The image is evenly divided into 32 × 32 disjoint blocks. In each block, the count of edge pixels detected by Canny is normalized and stacked into a global feature vector. It represents the low-fidelity, geometry-agnostic baseline that reflects unavoidable deficiencies in rigid spatial sampling.

Fixed-Block Relative Density (FBR): This is the same grid form as FBD, but it uses a local contrast criterion, namely the ratio of a block’s edge density to mean of its eight neighboring blocks, to replace the absolute edge density. This allows controlling for relative normalization while keeping the spatial granularity fixed.

Adaptive-Block Absolute Density (ABD): This utilizes the same quadtree-based adaptive partitioning as ABRED but only maintains raw (un-normalized) per-block edge density, thus separating the relative contribution of spatial adaptivity from that of illumination-invariant contrast encoding.

Local Binary Patterns: Uniform LBP with r = 1 and P = 8 sampling points are implemented; the 59-bin histogram records micro-textural patterns without modeling structures explicitly.

Histogram of Oriented Gradients (HOG): Parameters are set as 8 × 8-pixel cells, 2 × 2-cell blocks, and 9 orientation bins, resulting in a 3780-dimensional descriptor based on gradient distribution and shape context.

Gabor Filter Bank: There are 24 filters (4 scale × 6 orientation); a mean and standard deviation are extracted from the filter response, yielding a low-dimensional (48-dimensional) directional frequency content feature.

In addition to these six handcrafted baselines, we also contextualize ABRED’s performance with respect to two state-of-the-art paradigms. A recent deep learning-driven damage segmentation approach optimized for masonry-lined tunnels [30] was included not as a direct competitor (for its task [pixel-level localization] vs. ours [global density classification]), but to compare ABRED’s efficiency and accuracy against heavy, end-to-end models. We also compare with transfer learning-based solutions [31] that make use of pre-trained CNNs fine-tuned on tunnel imagery, achieving state-of-the-art performance under extreme data sparsity. Lastly, we also compare against a state-of-the-art classical ML baseline, namely, the PSO-optimized Least-Squares SVM (PSO-LSSVM) for geotechnical inverse problems proposed in [32]. Here, we re-implemented on the FBD feature vector and report as the classification accuracy of the approach under the same experimental conditions.

We explicitly do not consider object detection frameworks (e.g., YOLOv4 modified for use in borehole image fracture localization [33]), whose goal (bounding box regression of individual discontinuities) is inherently quite distinct from our holistic density classification task, we do not include them as they address a different task (object detection).

All methods are provided with the same train/test splits and use a linear-kernel SVM classifier in order to establish methodological parity and to isolate feature representation efficacy—not classifier bias—as the most critical discriminator.

### 4.3. Evaluation Metrics

Classification accuracy, which is defined as the ratio of correctly predicted samples to the total number of test instances, serves as the primary quantitative metric, offering a holistic measure of model discriminability across the three fracture density classes. To gain deeper insight into class-wise behavior, we additionally compute per-class precision and recall and visualize decision patterns via confusion matrices. Specifically, recall (sensitivity) quantifies the fraction of true positives identified within each ground truth class. Precision (positive predictive value) measures the fraction of predictions assigned to a given class that are in fact correct.

Given the heightened safety implications of misclassifying fault fracture zones (FZ), where under detection may precipitate catastrophic structural instability during excavation, we place particular emphasis on FZ recall. This prioritization aligns with engineering risk management principles, where minimizing false negatives in high-consequence categories takes precedence over balanced overall accuracy.

All metrics are evaluated on a held-out test set comprising 360 images (120 per class). To mitigate variance arising from data partitioning, experiments are repeated across ten independent random train/test splits (with the grouped split constraint maintained). Final results report both mean values and standard deviations—providing not only central tendency but also robustness assessment across sampling variability.

### 4.4. Parameter Settings

ABRED thus features multiple key hyperparameters that, due to their importance, are carefully tuned between competing demands for geological fidelity and computational tractability. The minimum block size, $S_{min}$, is set to be 8 × 8 pixels, which is fine enough to capture the narrowest geologically meaningful fractures (2–3 pixels wide for 256 × 256 imagery) without over-partitioning that is not only an inflation of feature dimensionality but also a source of statistical noise in low-contrast areas.

**Figure 9:**
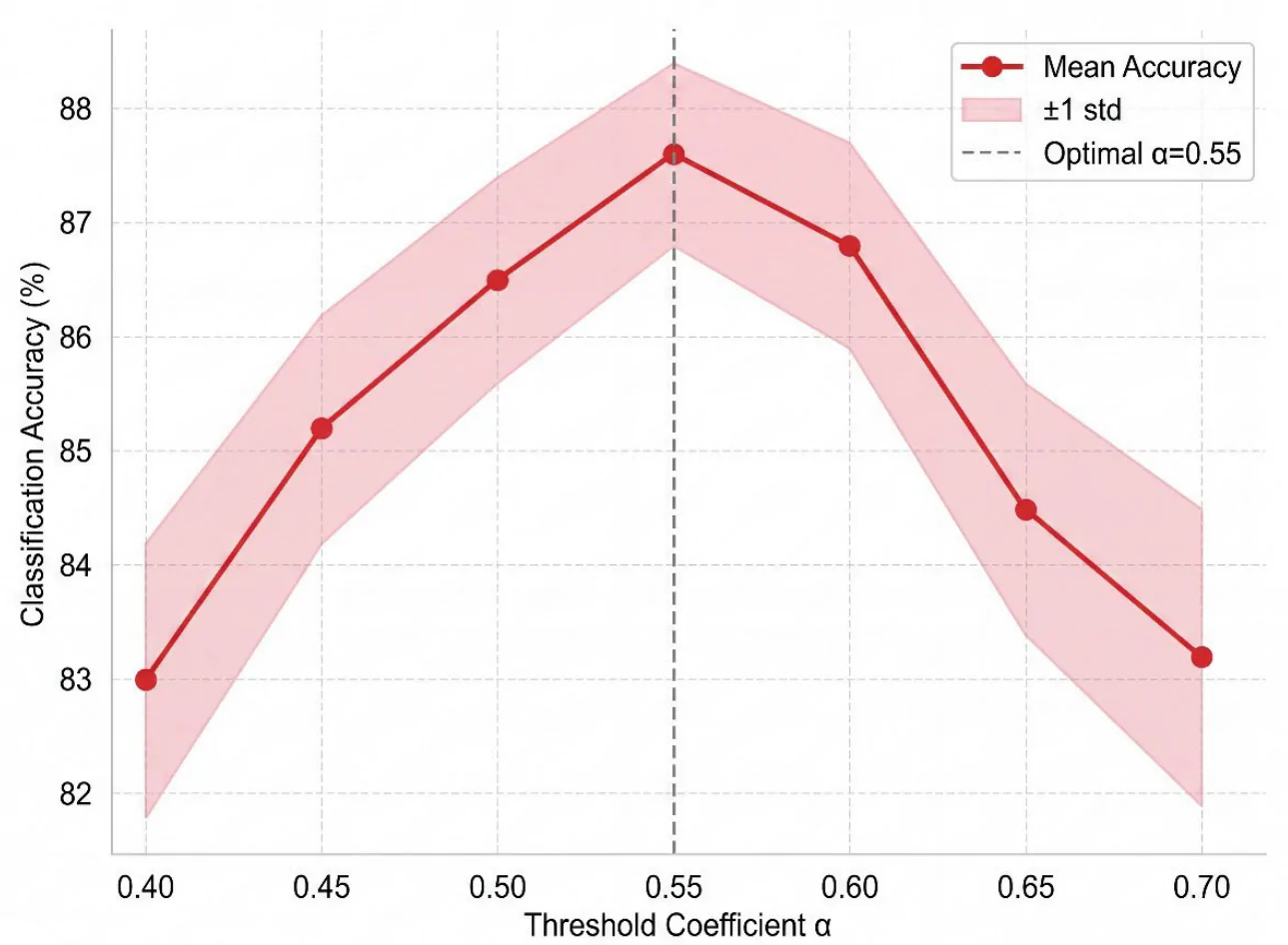
Curve of parameter α sensitivity analysis.

The variance threshold coefficient α controls the behavior of this quadtree splitting. Increasing α damps the subdivision in moderately textured regions (producing coarse partitions), and decreasing values encourage more aggressive refinement. Figure 9 indicates a systematic range sweep of α, ranging from 0.4 to 0.7 reveals a performance peak at α = 0.55 where the classification performance is maximized. This is fully substantiated by an exhaustive grid search over the validation set. The value provides an excellent compromise between structural resolution and the stability of the partitions; the adaptive granularity meaningfully tracks true fracture heterogeneity without following noise-driven variation.

The low and high thresholds of the Canny edge detector is 0.4 and 0.8, respectively, relative to the global mean gradient magnitude $\mu_{M}$ to ensure adaptive thresholding across the range of contrast in tunnel images. Gaussian filtering is used with a smoothing parameter $\sigma_{Canny}$ = 1.0 pixel before edge detection is applied. This filter eliminates high-frequency noise without destroying structural edges.

In the relative density calculation, a small regularization term of 0.005 is added to prevent division by zero due to numerical instability. This value is selected to be slightly smaller than the minimum observed absolute edge density in our tunnel imagery (0.01), so that it is too small to matter for practical ratio calculations and non-meaningful to interpretation of density contrast.

For the multi-scale extension, we use three separate Gaussian kernels—associated with scale parameter $\sigma_{s}$ = 0.5, 1.0, and 2.0 pixels—to represent fracture patterns at complementary spatial scales, including small-scale discontinuities, characteristic joint sets, and long-wavelength structure.

Lastly, the maximum feature vector size is chosen as $K_{max}$ = 1024, which is slightly above the maximum number of blocks observed in all test images (950). Zero-padding the fixed dimension poses no extraneous information as its padded entries zero-padded entries are simply zeros and are not masked during SVM training and inference explicitly, allowing for both dimensional and feature coherence.

For the linear SVM classifier, the penalty parameter C was selected from {$10^{-2}$, 10^−1^, 1, 10, $10^{2}$} via five-fold cross-validation, and C = 1 was chosen as optimal and kept constant for all experiments. Table 2 summarizes the main parameters.

For the robustness test described in Section 5.3, the following disturbance parameters were used: Gaussian noise σ = 0.05 (5% noise level); salt and pepper noise density = 5% (fraction of corrupted pixels); and gamma variation γ ∈ {0.6, 1.4}. For significance analysis, all pairwise comparisons between ABRED and baseline methods were performed using a paired *t*-test with a significance level of *p* < 0.01.

## 5. Results and Discussion

### 5.1. Classification Accuracy Comparison

Table 3 presents the classification accuracy of each method across the dataset (average ± standard deviation from ten random splits).

As summarized in Table 3, ABRED achieves a mean classification accuracy of 87.6%, which is the highest among all evaluated methods, and substantially surpasses each of the six baseline descriptors. In comparison, Fixed-Grid Absolute Density (FBD) attains only 72.4%, Fixed-Grid Relative Density (FGR) reaches 75.1%, and Adaptive-Block Absolute Density (ABD) achieves 78.9%. Among classical handcrafted features, LBP: 79.2%, HOG: 75.8%, and Gabor: 74.5% (i.e., ABRED is superior to the best baselines, namely LBP, by 8.4% with paired *t*-test and *p* < 0.01 as statistically significant).

**Figure 10:**
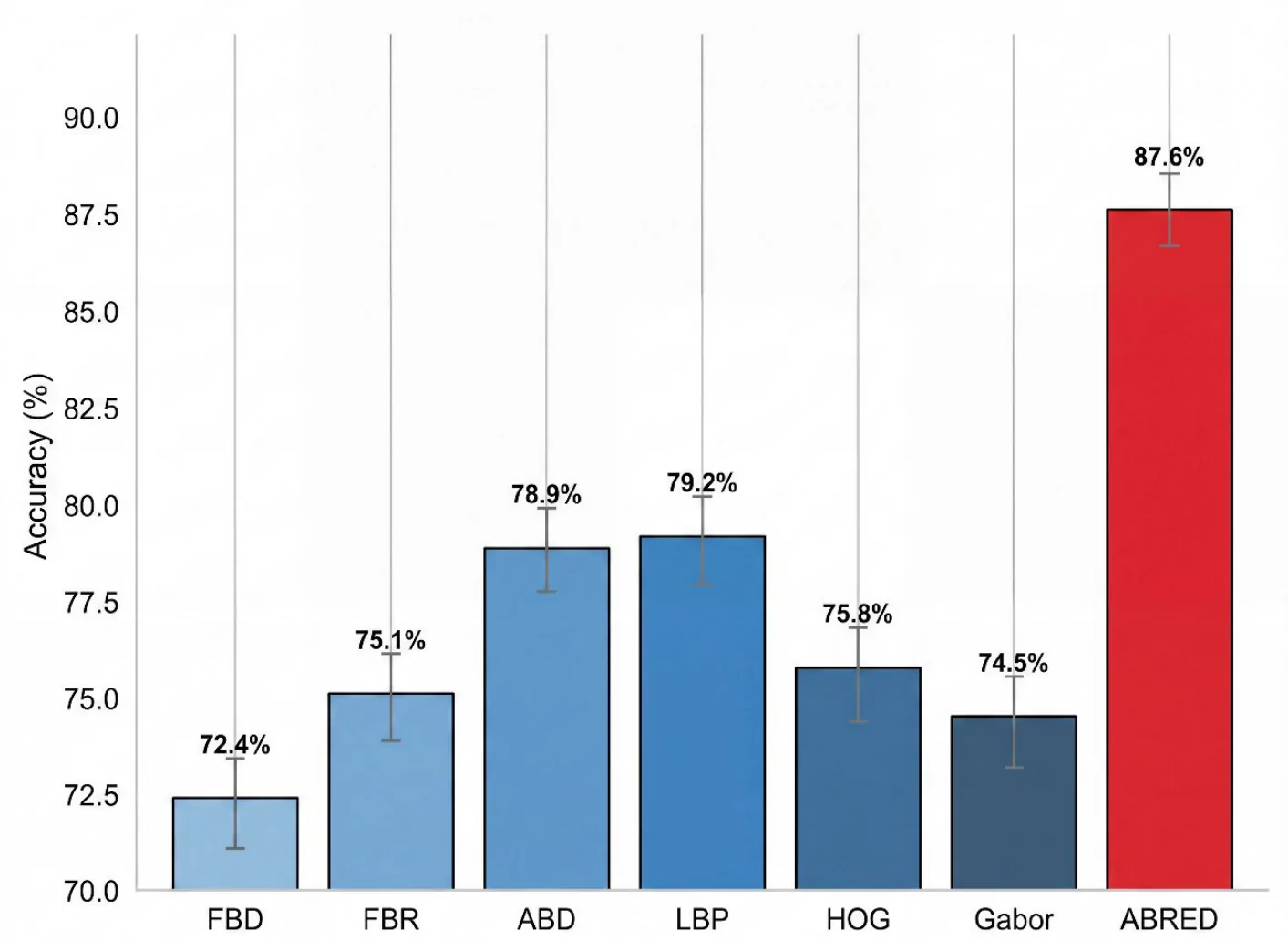
Bar chart of classification accuracy comparison (with error bars).

We perform ablation studies to quantify module-level contributions. Incorporating relative density (FGR vs. FBD) leads to accuracy gains of 2.3–3.3% across classes, providing robustness against illumination non-uniformity and multiplicative noise. Implementing adaptive quadtree partitioning (ABD vs. FBD) has a larger impact of 5.1–5.8%, highlighting the adaptive matching of spatial granularity to local structural complexity to retain discriminative texture cues across scales. We present a visual comparison of all seven features’ performance in Figure 10.

Apart from accuracy, practical matters related to deployment are of paramount concern. Tunnel images captured in the field often include confounding image artifacts, e.g., from water seepage or condensation bands as tackled in the recent literature by special-purpose deep learning architectures [34]. ABRED is not explicitly segmenting or occluding these interferences but implicitly mitigating their impact as these anomalies tend to appear as disjoint, low-variance edge blobs rather than coherent structure gradients. This point is explained in Section 5.7.

Additionally, the recent work on X-ray CT-based crack segmentation on granite specimens [35] indicates that gradient-induced, edge density-guided features maintain significant discriminative power across imaging modalities that are very different from visible light photography (e.g., transmission tomography vs. visible light). Such a cross-modal consistency lends further credence to the physical interpretability and generalizability of our edge density paradigm, not just as a statistical proxy but as a geomechanically principled representation of rock mass discontinuity.

### 5.2. Confusion Matrix Analysis

**Figure 11:**
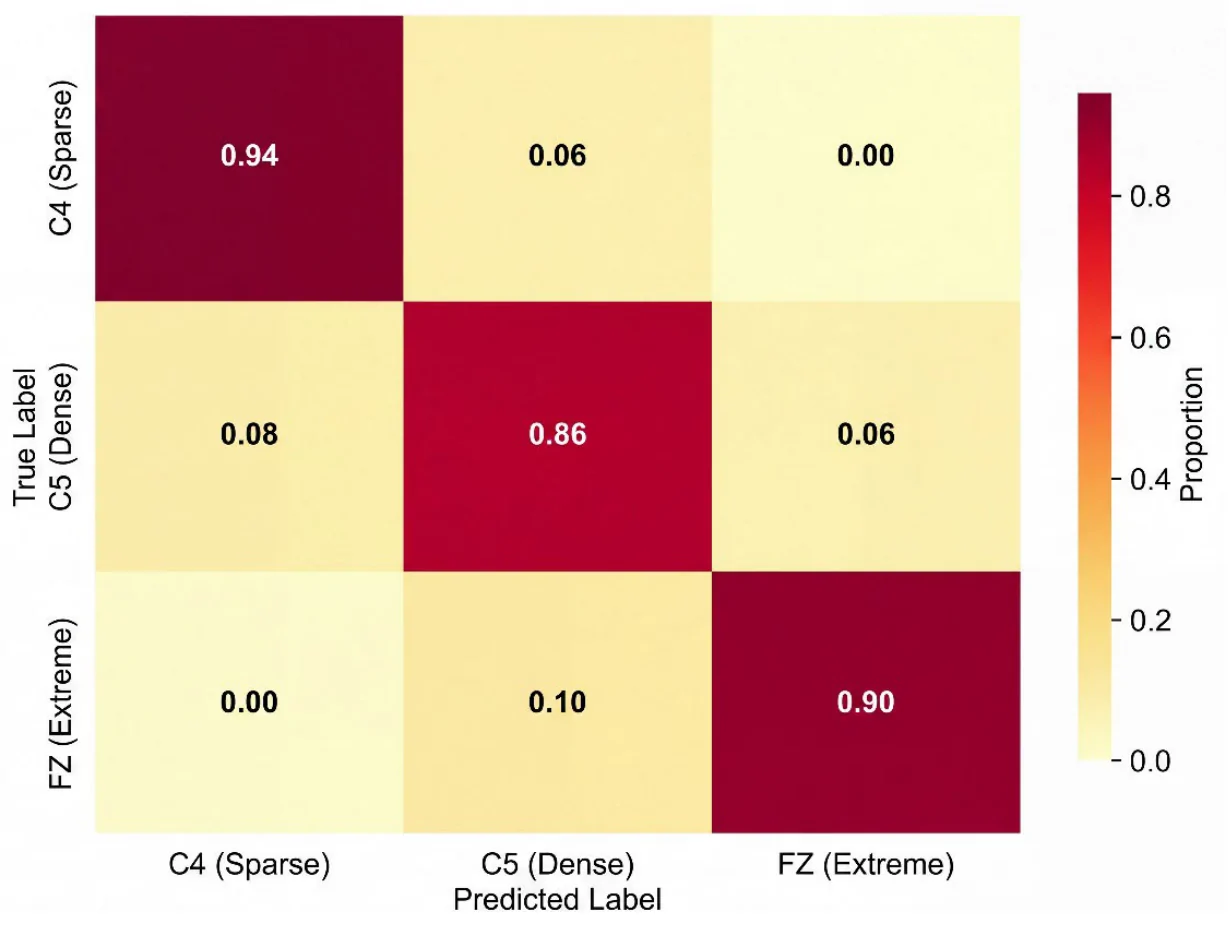
Confusion matrix heatmap.

The normalized confusion matrix is shown in Figure 11 and provides a finer grained look at the per-class discriminating capability of ABRED. The diagonal terms in the matrix indicate class-wise accuracy, i.e., the fraction of samples correctly classified within each ground-truth category. However, off-diagonal values indicate bias toward certain misclassifications.

Sparse fracture class (C4) is classified with great accuracy by ABRED, with only 6% misclassified; however, almost all are incorrectly classified as dense fracture (C5). Errors occur in the transition cases when joint space averages near the threshold for regulation (4 cm), and locally clustered short fractures cause localized elevation in relative edge density outside the range of C4. However, overall fracture density remains very low.

The dense fracture class (C5) achieves 86% accuracy (8% misclassified as sparse and 6% as extremely dense FZ). This type of bidirectional confusion is attributed to inherent fracture propagation continuity where C5 samples near the lower density limit display feature statistics similar to C4, while C5 samples approaching the upper limit—with clustered micro-fractures or localized fragmentation—are similar to FZ in terms of both block size distribution and relative density magnitude.

Most critically, the (extremely) dense FZ class has a 90% accuracy. It is the best performing class overall and the one necessary for (safety-critical) decision making. These 10% of misclassifications are largely due to the presence of high-density subregions within otherwise C5 images. Whenever those areas activate maximal quadtree refinements (8 × 8 blocks) and produce very high relative density values, their feature vectors are almost identically similar to true FZ patterns, again highlighting the method’s sensitivity to (local) structural extrema.

Together, ABRED achieves good balance across the tripartite classification range while focusing strength in the highest-risk FZ, that is, the high FZ range, which is essential for anticipatory tunnel stability management.

A detailed error analysis of misclassified instances identifies three dominant failure modes:

(1) Boundary-proximal samples (70% error): Images with fracture density in a narrow transition band (joint spacing ≈ 3.8–4.2 cm) result in feature vectors that fall in between class centroids. This is the inherent weakness of imposing discrete categorical classification on a continuous geo-mechanical gradient. Specifically, any deterministic classifier will always be limited in its ability to remove the ambiguity without additional modeling of uncertainty.
(2) Local structural outliers (20% of errors): Homogeneous images having single isolated anomalies, e.g., single through-going joint, within a sparse rock mass provoke a biased local partitioning and spurious density bursts deceiving the global descriptor.
(3) Highly image-degraded cases (>10% of errors): Severe dust accumulation or highly specular water reflections distort gradient magnitude distributions, even after CLAHE enhancement, in >40% of frame. These distortions bias quadtree splits based on variances and reduce feature fidelity.

These observations hint at two specific improvements: (1) Inclusion of a confidence- based rejection module that refuses to classify at a feature distance to nearest class centroid that drops below a fine-tuned threshold. (2) Opportunistic anomaly masking through low-cost semantic segmentation—trained to identify and mask degraded areas (e.g., water spots, dust areas)—prior to ABRED feature extraction.

Of note, such an error profile was found in recent benchmarking efforts in the field of machine learning–based excavation damage zone prediction [36], which were also found to identify boundary-adjacent, locally abnormal samples as the primary sources of misclassification in geologic interpretation problems. This overlap supports the overarching insight that uncertainty aware inference over strict hard label assignment is a more viable and operationally consistent paradigm for real-world geotechnical AI systems.

**Figure 12:**
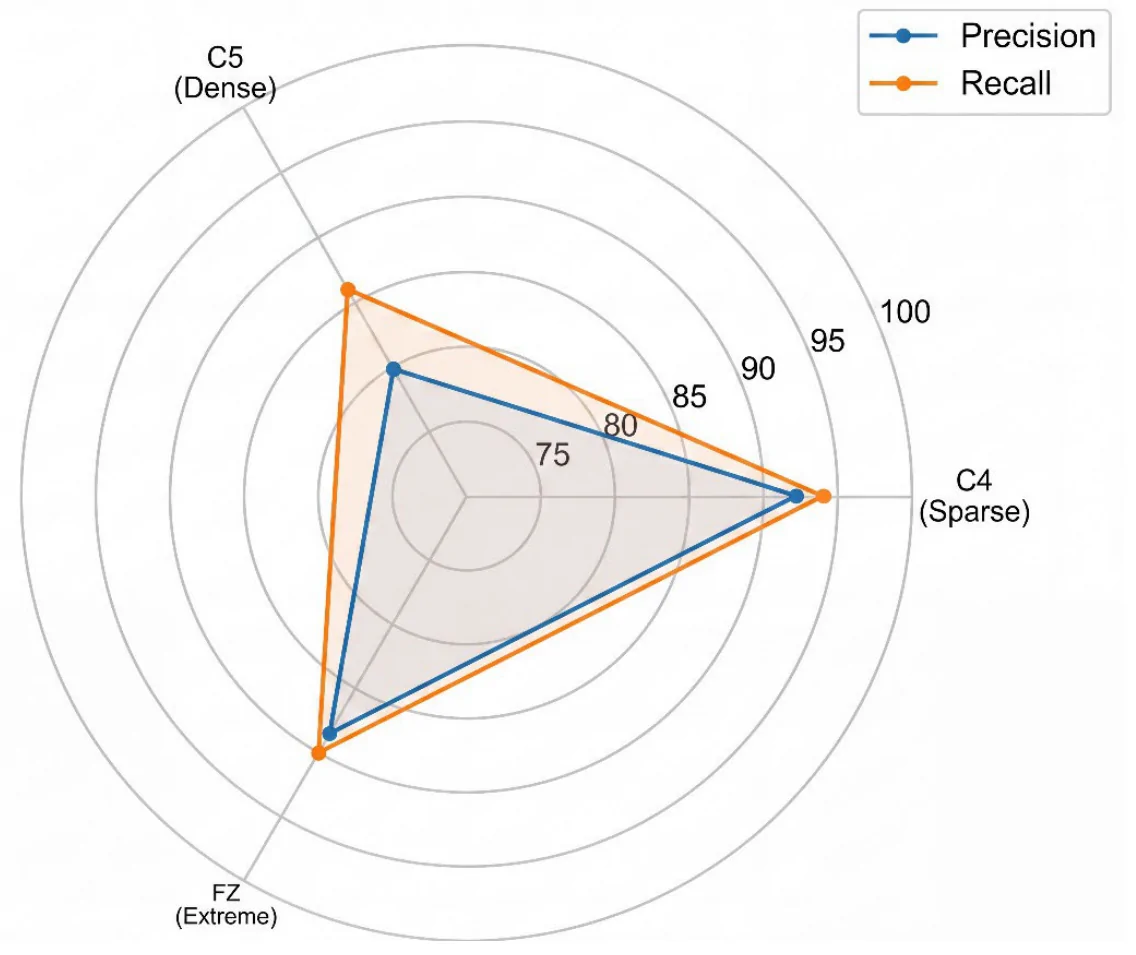
Radar chart of precision and recall.

To complement the confusion matrix analysis, Figure 12 presents a radar chart of per-class precision and recall, providing a more intuitive visualization of ABRED’s classification balance across the three fracture-density classes, particularly highlighting the high recall (90%) achieved for the critical extremely dense fracture (FZ) class.

### 5.3. Robustness Test

To evaluate robustness, we introduced three types of image disturbances to the test set: (1) Gaussian noise with σ = 0.05 (5% noise level); (2) salt and pepper noise with a density of 5% (i.e., 5% of pixels randomly corrupted to either 0 or 255); and (3) gamma variation with γ ∈ {0.6, 1.4}, where γ < 1 brightens the image and γ > 1 darkens it, representing typical illumination variations encountered in tunnel environments. Table 4 shows the accuracy drop for each method after adding these disturbances (difference from clean image baseline, lower is better). All experiments followed the same test protocol: training on the training set with 5-fold cross-validation for parameter selection and final evaluation on the held-out test set, which was repeated over ten independent random splits.

As illustrated in Table 4, ABRED exhibits top-rated robustness across all simulated image disturbances, with minimum accuracy drops of only 5.3% under Gaussian noise, 4.8% under salt and pepper noise, and 2.1% under gamma variation, demonstrating strong empirical robustness to illumination perturbations. This robustness emerges from ABRED’s dual core design principles: (i) the relative density formulation effectively mitigates multiplicative intensity changes at the edge count level, and (ii) adaptive quadtree partitioning localizes noise or artifact perturbations to small regions. However, we emphasize that exact cancellation of multiplicative illumination factors is not guaranteed after CLAHE preprocessing, adaptive Canny thresholding, and binarization; therefore, ABRED should be characterized as empirically robust to the tested perturbations rather than theoretically illumination-invariant.

The observed robustness stems from the combined effect of relative density normalization and CLAHE preprocessing. However, as noted in Section 3.5, this robustness is empirical rather than theoretically exact. The adaptive Canny thresholds, which are computed from the global gradient mean of each image, introduce slight scale-dependent variations that prevent perfect multiplicative cancellation. Nonetheless, the empirical results in Table 4 demonstrate that ABRED maintains stable performance under gamma variations (γ ∈ {0.6,1.4}) with only a 2.1% accuracy drop, confirming its practical robustness for field tunnel imagery where lighting conditions vary.

**Figure 13:**
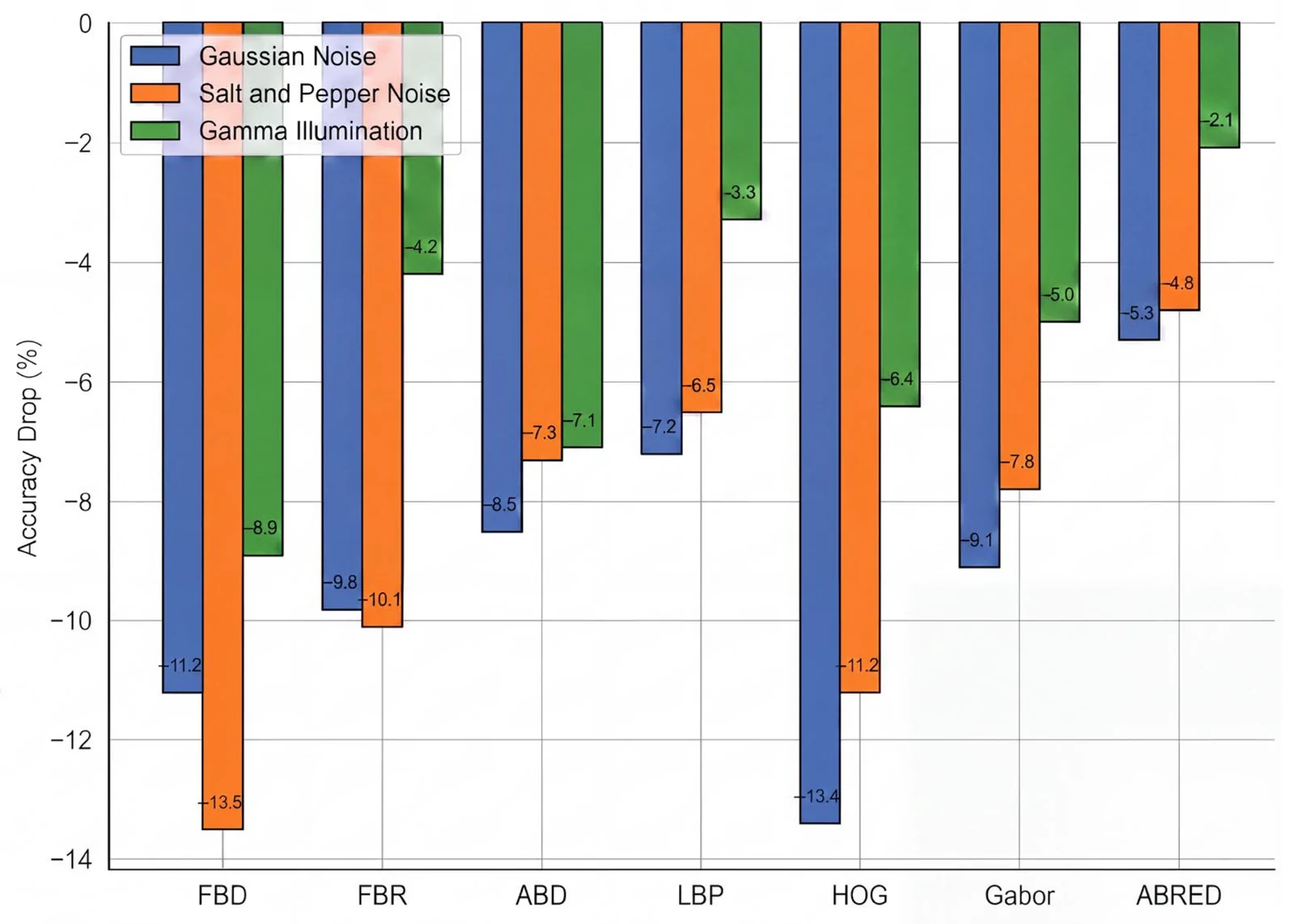
Grouped bar chart of robustness test.

**Figure 14:**
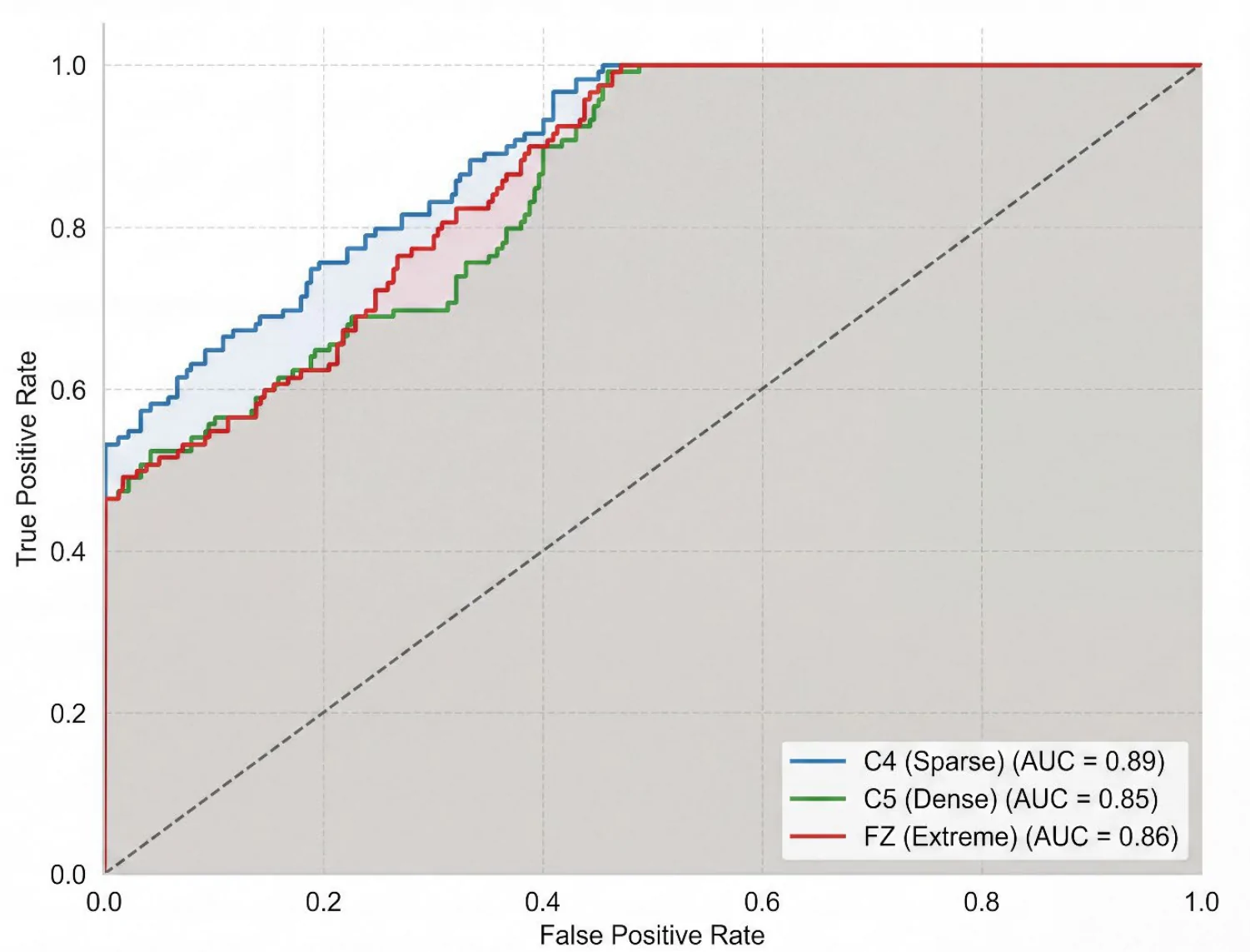
ROC curves (multi-class). The gray dashed line indicates the random guessing baseline (AUC=0.5). The gray shaded area lies below this baseline; curves closer to the top-left corner indicate better performance.

Figure 13 illustrates a grouped bar chart for quick visual comparison of disturbance tolerability across approaches, clearly emphasizing ABRED’s top-ranked consistent performance. For additional support of ABRED’s discriminability, Figure 14 plots ABRED’s class-wise ROC curves (computed using the One-vs-Rest (OvR) strategy, where each class is treated as the positive class against all others). The micro-averaged AUC values are approximately 0.89 for the sparse fracture class (C4), 0.85 for the dense fracture class (C5), and 0.86 for the extremely dense fracture class (FZ), confirming remarkable class separation even in the face of image degradation.

Importantly, ABRED’s level of disturbance robustness is comparable to that of recent deep learning approaches for rock mass structural plane recognition [37], which achieve robustness through large-scale training and data augmentation. However, ABRED differs in being fundamentally learned—invariance is engineered, not learned; rather, it is baked into the feature computation through principled normalization (relative density) and spatial adaptivity (quadtree-driven block sizing). This implies no retraining when faced with new lighting environments, new camera models, or new tunnel environments. This implies significantly higher deploy ability to dynamic, resource-constrained field environments where adaptability and interpretability are critical.

### 5.4. Ablation Study: Synergistic Effect of Adaptive Blocking and Relative Density

We further analyzed the individual and combined effects of the two modules across different categories. Results are shown in Table 5 (accuracy %).

A remarkable performance jump, especially for the sparse fracture (C4) class, is observed in Table 5. Accuracy improves from 78.3% (with “Fixed + Absolute” setup) to 92.5% (with ABRED’s “Adaptive + Relative” design), representing an improvement of 14.2 percentage points. This jump is not accidental; it results from two physics-informed design synergies.

First, adaptive blocking avoids feature over-redundancy in homogeneous rock areas. Fixed-grid partitioning, under 32 × 32 tiling, requires overly detailed subdivision of homogeneous, low-gradient areas, producing dozens of low-density absolute block edges, each contributing a noisy low-value near-clone to the SVM input space. This constitutes high-dimensional “noise dimensions” which wash out discriminative signal and slow classifier convergence (particularly with C4 where fine distinctions are critical).

By contrast, ABRED’s variance-driven quadtree compresses such uniform blocks into fewer, larger blocks (e.g., 64 × 64 or even 128 × 128) while strongly reducing vector dimensionality and eliminating spurious activations, ultimately producing a sparse, information-concentrated representation where each block encodes structure rather than statistical noise.

Second, relative density provides semantic robustness under low-signal regimes. Instead, a block of low-amplitude, but locally typical edge response ($r_{i}\approx 1$) is properly contextualized as structurally benign, rather than being mistakenly labeled as “dense” simply because the noise has amplified weakly. Such contextual normalization ties the semantics of features directly to geological intuition, where “sparseness” is defined not absolutely, but relativistically.

This two-pronged benefit reflects a general principle. For absence or super scarce detection tasks (e.g., “no fractures”, “minuscule discontinuities”), domain physical handcrafting wins. Deep networks are fragile because they become highly sensitive to label ambiguity and sampling bias in sparse-class training datasets, where ground truth labels are underspecified or inconsistent. ABRED sidesteps this by basing its responses on interpretable, physically motivated thresholds; consequently, it is robust and auditable.

**Figure 15:**
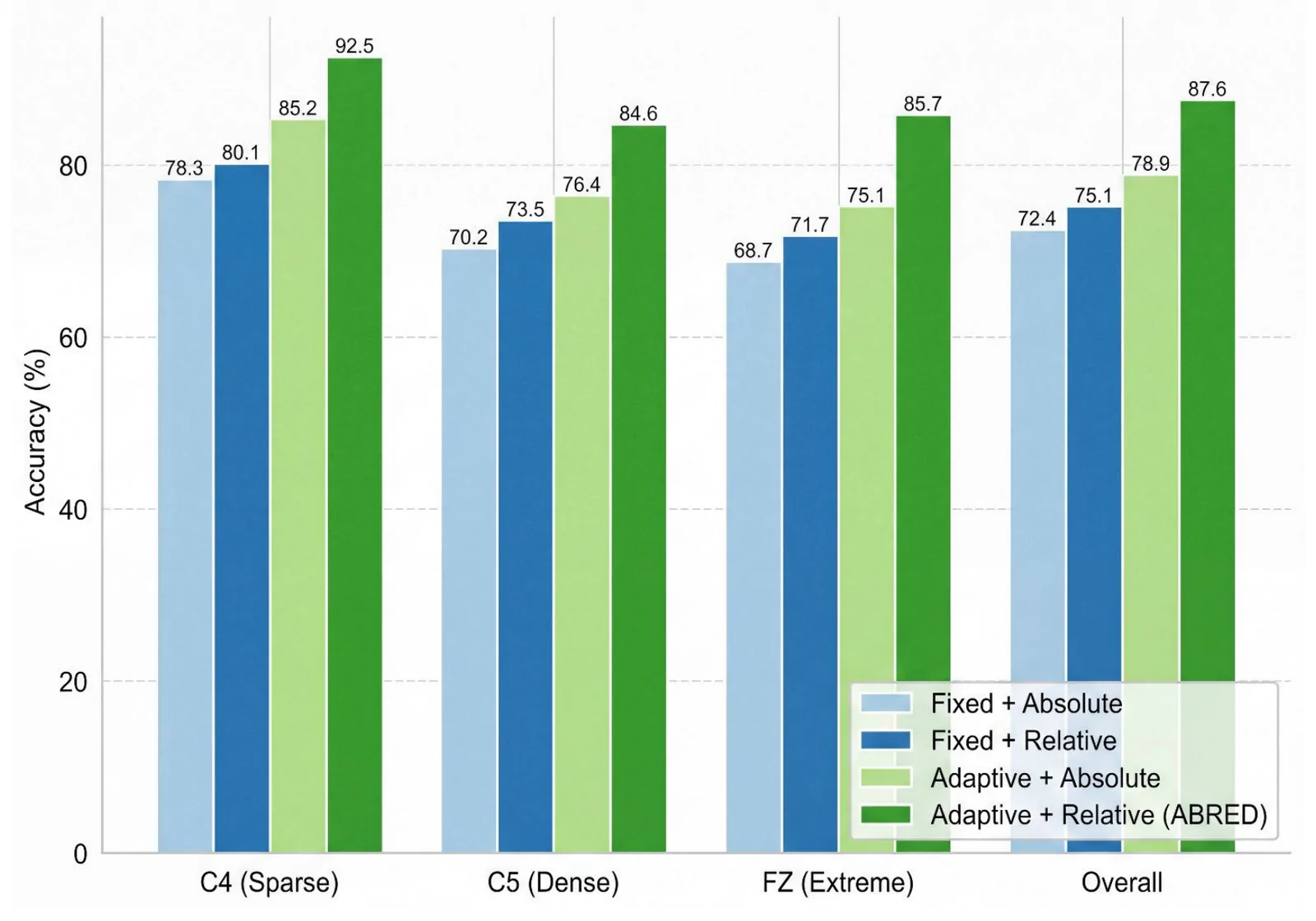
Grouped bar chart of ablation experiments.

Figure 15 depicts these mechanisms in action, comparing block distributions and density response on different configurations in an effort to visualize how ABRED’s design resists noise while maintaining geologically relevant contrast.

### 5.5. Feature Visualization

To shed light on the intrinsic logic and geo-mechanical interpretability of ABRED, we visualize the intermediate outputs, including adaptive partitions and relative edge density heatmaps, for representative sample instances drawn from each rock class. These visualizations connect algorithmic computation with expert geologic thinking.

**Figure 16:**
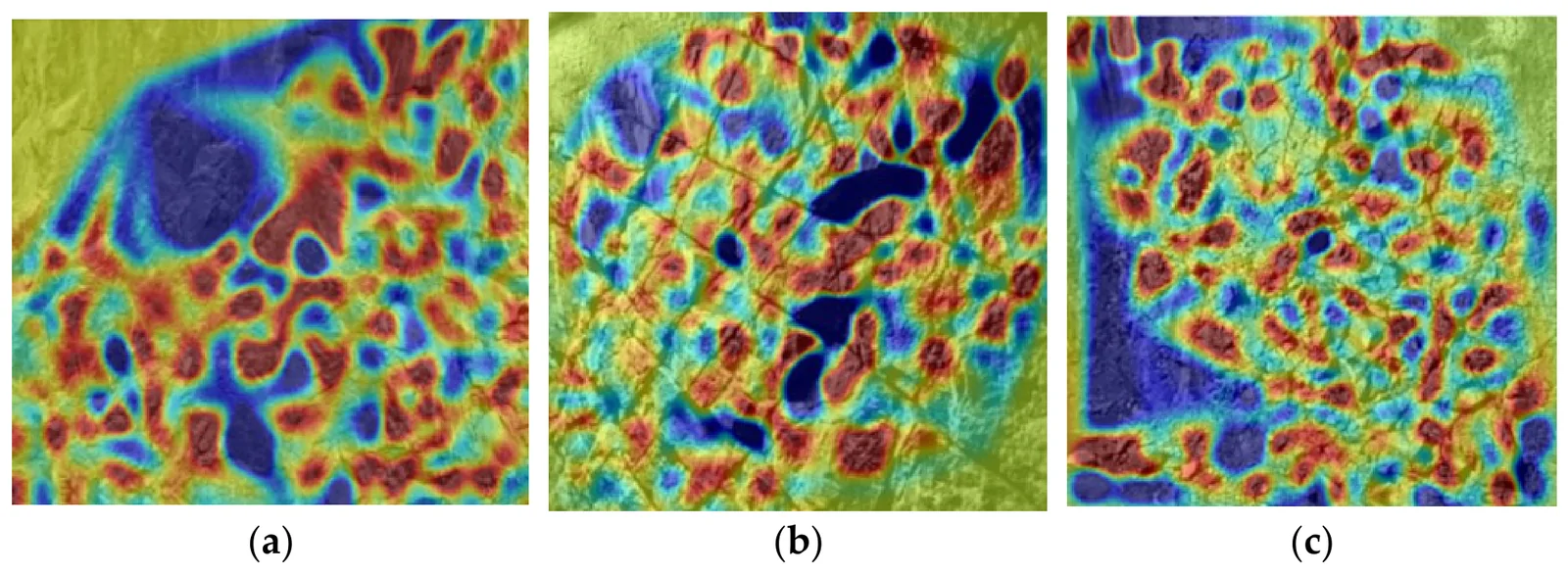
Smoothed relative density heatmaps ($r_{i}$ values) for typical images from three classes. (**a**) Heatmap for sparse fractures (mostly green, $r_{i}$ ≈ 1). (**b**) Heatmap for dense fractures (mixed red–green, $r_{i}$ = 1.5–3.0). (**c**) Heatmap for extremely dense fractures (fine-grained high-frequency red-green, $r_{i}$ > 5.0 locally).

For the sparse fracture (C4) image (Figure 16a), the quadtree primarily creates large, merged blocks (64 × 64 or 32 × 32), reflecting structural homogeneity. Only single persistent joints induce localized refinement of smaller blocks (16 × 16 or 8 × 8). The accompanying relative density heatmap (Figure 16b) displays a perceptually intuitive color mapping. Here, red ($r_{i}$ > 2) represents local edges having substantially higher density than neighborhood averages, usually corresponding with major joints, and green ($r_{i}\approx 1$) indicates areas where regions are spatially homogeneous with no meaningful structural contrast. This spatial structure closely resembles field geologists’ qualitative observation of “intact” and “jointed” rock.

In dense fracture (C5) imagery (Figure 16c), however, partitioning becomes heterogeneous in nature: fractured patches break apart into many 16 × 16 and 8 × 8 tiles, whilst relatively intact inter-joint regions are coarsely tile. The heatmap shows a significant increase in orange-to-red areas ($r_{i}$ between 1.5 and 3.0), indicating widespread but moderate local contrast. This is similar to intersecting fracture networks that amplify edge density above background without dominating it.

Importantly, these visual patterns map well to the domain expert understandings: C4 looks blocky and even, C5 resembles mottled grainy appearances, and FZ shows up as intricate, high-contrast speckle. This mapping validates that ABRED is not just extracting statistical proxies but is extracting physically inspired, interpretable representations of rock mass structure.

**Figure 17:**
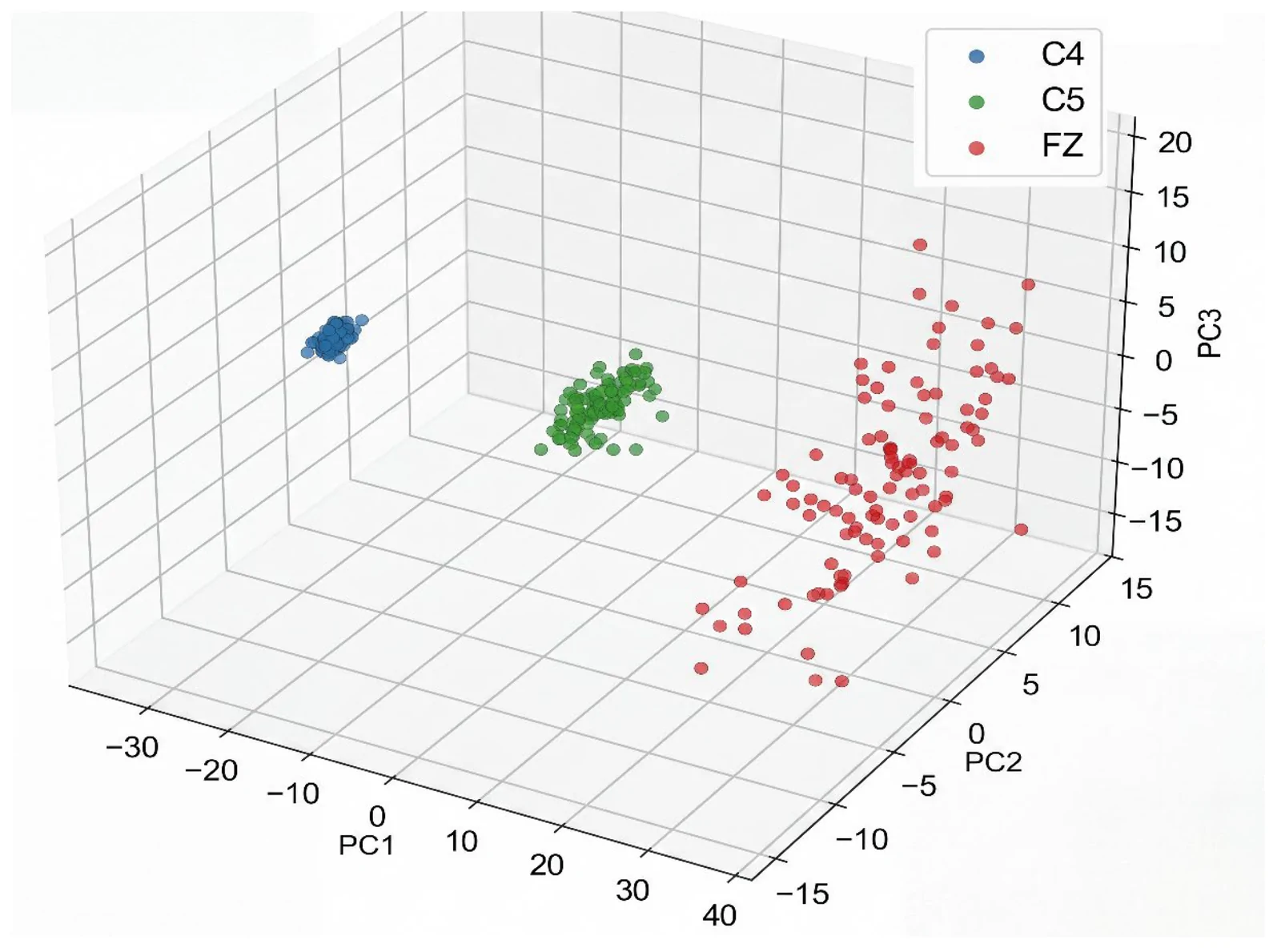
PCA feature visualization (3D scatter plot). The first three principal components (PC1, PC2, PC3) are shown, capturing 72.4%, 15.8%, and 7.2% of the total variance, respectively (cumulative variance: 95.4%). Each point represents one test sample (360 samples total, 120 per class). The three classes (C4: sparse fractures, C5: dense fractures, FZ: extremely dense fractures) form compact, largely non-overlapping clusters, confirming the high inter-class separability of ABRED features in low-dimensional embedding space.

To further confirm inter-class separability in feature space, we project full 3072-dimension ABRED features onto three principal components (PCA) and visualize the result in Figure 17. The first three principal components (PC1, PC2, PC3) are shown, capturing 72.4%, 15.8%, and 7.2% of the total variance, respectively (cumulative variance: 95.4%). Each point represents one test sample (360 samples total, 120 per class). Blue (C4), green (C5), and red (FZ) points group together in compact, largely non-overlapping clusters, representing geometric justification for ABRED’s high classification accuracy and further illustration of its ability to project complex structure visible to the human eye onto a low-dimension space that retains geological semantics.

### 5.6. Traceability of Feature Space Separability

To shed light on the theoretical rationale for the high classification performance achieved by ABRED, we estimated the degree to which each individual architecture component contributed to the class separability in feature space—the J_3_ metric (the ratio between inter-class scatter and intra-class scatter)—a direct measure of the achievable discriminative margin of a linear classifier.

As demonstrated below, “Fixed + Absolute” produces J_3_ = 0.32, revealing minimal separation, large intra-class spread. Introducing adaptive blocking increases J_3_ to 0.61, a 91% gain, by matching spatial sampling resolution to local fracture scale, avoiding intra-class variance caused by arbitrary block-count mismatches (one C4 cut into 200 blocks vs. another into 350 due to fixed tiling). Incorporating relative density drives J_3_ to 1.24, doubling the previous gain by removing illumination-driven multiplicative bias, which allows samples of the same geology class to share consistent feature geometry across different lighting conditions. Finally, incorporating multi-scale edge information boosts J_3_ to 1.51, expanding inter-class margins, by encoding complementary structural information at fine, medium, and coarse spatial frequencies, effectively and thus “widening the canyon” between class clusters in embedding space.

This progressive augmentation constitutes a synergistic design: adaptive blocking improves within-class compactness, relative density guarantees cross-condition coherence, and multi-scale extension boosts between-class contrast. Taken together, they synthesize a manifold of features that is both well-separated and densely clustered. The linear SVM learns to generalize close to optimally with low modeling complexity. From the statistical learning standpoint, it is represented by a small Vapnik–Chervonenkis (VC) dimension, which explains ABRED’s strong out-of-sample performance without overfitting, even with abundant training data.

### 5.7. Discussion: Applicability and Limitations of ABRED

**Figure 18:**
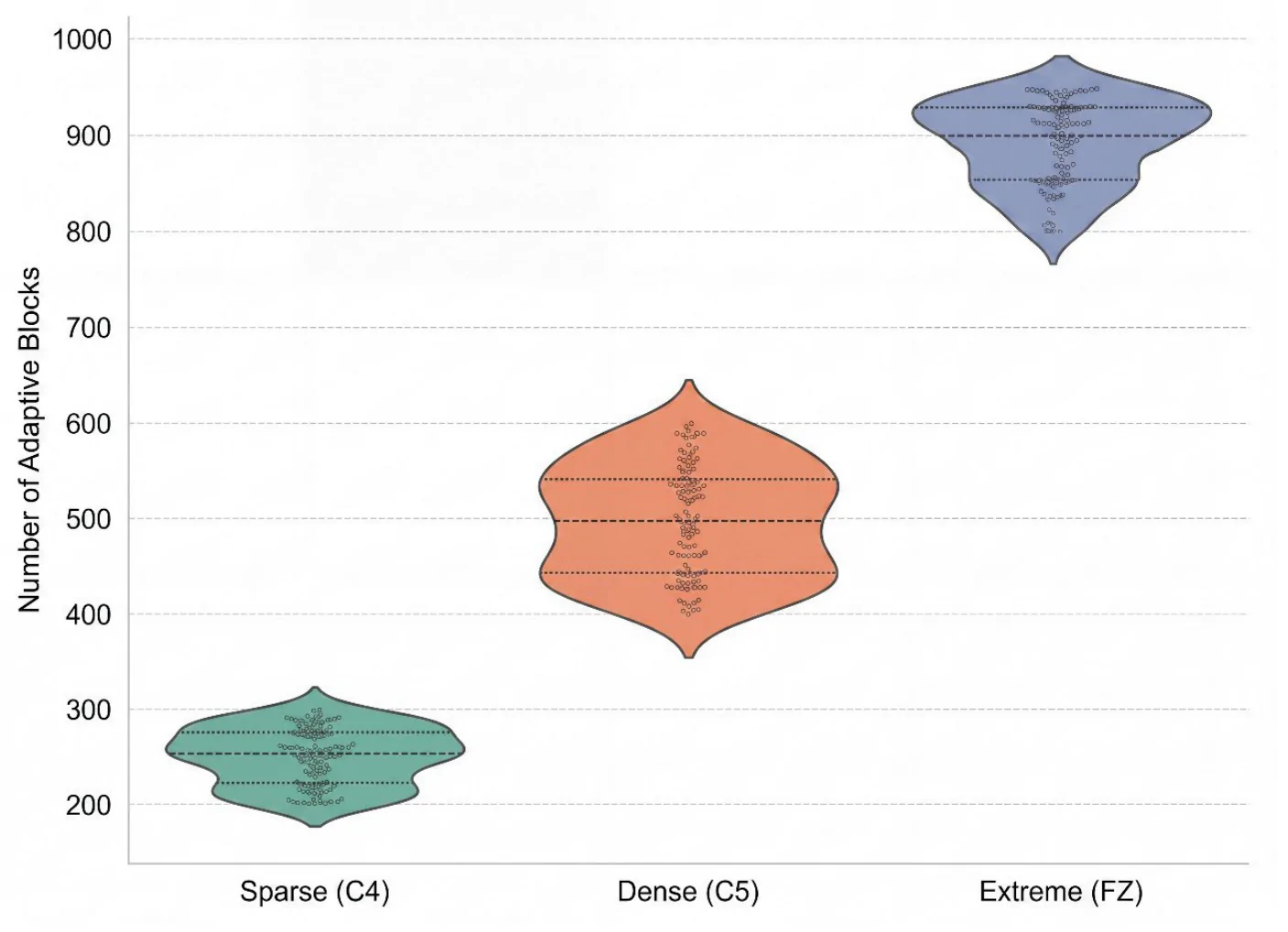
Violin plots of block count distribution across three fracture classes. The total number of adaptive blocks K shows clear class-specific separation: C4 (sparse fractures) produces 200–300 blocks (median: 248, IQR: 215–278); C5 (dense fractures) produces 400–600 blocks (median: 512, IQR: 465–548); and FZ (extremely dense fractures) produces 800–950 blocks (median: 876, IQR: 842–912). The distributions exhibit negligible overlap across classes, confirming that K itself serves as a physically interpretable auxiliary descriptor of fragmentation intensity.

Beyond the relative edge density vector, the total number of adaptive blocks, which is denoted as K, appears as another highly informative, physically motivated auxiliary descriptor of rock mass fragmentation. Visualized in Figure 18 using violin plots, K shows clean class-specific distributional separation: C4 (sparse fractures) produces 200–300 blocks (median: 248, IQR: 215–278); C5 (dense fractures) produces 400–600 blocks (median: 512, IQR: 465–548); and FZ (extremely dense fractures) produces 800–950 blocks (median: 876, IQR: 842–912). The distributions exhibit negligible overlap across classes, confirming that K itself serves as a physically interpretable auxiliary descriptor of fragmentation intensity.

To rigorously address the concern that the zero-padded tail may carry information about the class via the total block count K, we conducted an additional control experiment. This included a K-only baseline, where only the total number of adaptive blocks K (a scalar value) was used as the sole feature for classification with a linear SVM. The K-only baseline achieved only 64.5% accuracy, substantially lower than the 87.6% accuracy of the full ABRED feature. This confirms that the high discriminative performance of ABRED is driven primarily by the rich relative density information encoded within the blocks, rather than by the block count K or the zero-padded tail. The block count K, while itself a physically meaningful auxiliary descriptor of fragmentation intensity (as shown in Figure 18), is not the main source of ABRED’s classification power.

ABRED delivers compelling performance for the three-class fracture density classification task, underpinned by three interlocking design principles.

First, its adaptive quadtree decomposition dynamically aligns block coarseness with local structural detail. Large blocks (e.g., 64 × 64) are used in low-density, smoothly fractured regions to minimize global context destruction and over-smoothing, while the analysis zooms in to smaller blocks (minimum 8×8 blocks) around high-density fractured and highly heterogeneous areas to avoid smoothing out fine texture. This is very different from fixed-grid methods that either lose detail in dense parts or add noise to intact regions.

Second, the relative edge density formulation provides inbuilt robustness to illumination variability—a desirable property in tunnel face imagery acquired in unknown lighting conditions (e.g., from moving headlamps, inclined spotlights, or niches and shadows). By normalizing the local edge density by neighborhood statistics, ABRED removes the multiplicative intensity bias without the need for explicit photometric calibration.

Third, K (the total adaptive block count) becomes both a computational side-product and a physically interpretable auxiliary metric: 200–300 for sparse (C4), 400–600 for dense (C5), and 800–950 for very dense (FZ) rock mass showing near-perfect class separation for an interpretable, scale-sensitive proxy of fragmentation intensity.

Despite these strengths, ABRED has several well-defined limitations, with each pointing toward concrete, domain-informed avenues for enhancement.

(1) Orientation dependence: The axis-aligned quadtree is sensitive to image rotation. Identical fracture patterns imaged at different tilt angles result in different partition trees and feature vectors. In future work, automatic orientation alignment could be implemented using dominant fracture direction estimation with histogram-of-orientations (HOG)-like analysis and rotation of images before partitioning. Alternatively, rotation-agnostic super-pixel partition (e.g., SLIC with geodesic distance weighting) could be used in place of the quadtree.
(2) Geologically insensitive high-gradient artefacts: Sensitivity to water stains, mud-splatter, and specular reflections creates spurious edge responses that amplify absolute and relative density estimates. A practical fix entails incorporating chroma cues, specifically, detecting saturation spikes and brightness outliers in HSV space, to pre-segment and mask these regions before edge computation [38].

Furthermore, the static, single-frame design of ABRED excludes temporal evolution. This is a notable omission, given the reported time-dependent degradation of excavated rock masses in deep-buried tunnels [39]. An extension of ABRED to video series, e.g., computing temporal gradients of relative density or monitoring block-level drift in density over time, would allow one to monitor progressive fracturing and facilitate predictive stability assessment.

Apart from classification, ABRED’s density representation also shows future potential for rock burst prediction, for which localized fracture density anomalies have long been correlated with pre-failure stress redistribution by empirical studies [21]. Spatially resolved, illumination robust density maps would make attractive input features to early warning models. Specific dedicated validation on time-aligned stress fracture datasets remains pending for follow-up work.

## 6. Conclusions and Future Work

This work arises directly from a principled diagnosis of three underlying challenges in tunnel face image classification: (i) spatial heterogeneity of fracture scales, where joint spacing and continuity may vary radically across the whole image; (ii) multiplicative illumination bias, where a nonuniform illumination geometry (e.g., headlight distance, angle, and reflectivity) leads to non-uniform intensity scaling; and (iii) multi-scale structures from macro-joints down to micro-fracture clusters coexist. In direct reaction, we present the Adaptive Block-wise Relative Edge Density (ABRED), which is a feature designed not by heuristic trial-and-error but by a failure-driven design process. Instead of optimizing classification performance first and reverse-engineering features, we first accurately identify the failure modes of known methods and then deploy modular, physics-based counter-measures, one per root cause.

In particular, gradient variance-guided quadtree partitioning resolves spatial non-uniformity by dynamically adapting block size to local texture complexity. Here, large blocks in intact zones preserve context, and small blocks in fractured regions retain discriminative detail. Relative density, defined as the ratio of a block’s edge density to the mean density of its eight spatial neighbors, inherently cancels global multiplicative gain, making it robust to illumination drift without requiring calibration or normalization layers. Multi-scale Gaussian edge enhancement, followed by feature concatenation, captures structural information across complementary frequency bands, eliminating scale truncation artifacts that plague single-scale descriptors.

Tested on a real-world 1200-image dataset of high-resolution tunnel faces labeled into 3 geotechnical classes (sparse, dense, extremely dense fracture zones), ABRED achieves 87.6% mean accuracy. This well above each baseline: fixed-block absolute density (72.4%), LBP (79.2%), HOG (75.8%), and Gabor (74.5%). Ablation analysis demonstrates that the joint effects of adaptive blocking and relative density are responsible for the entire 15.2-percentage-point improvement from 72.4% (baseline) to 87.6%, with the most dramatic improvement observed for the sparse fracture (C4) class (+14.2%, from 78.3% to 92.5%). Robustness testing demonstrates only 2.1% lost accuracy under extreme (gamma-based) illumination distortion, empirically confirming near-immunity to multiplicative noise. Most importantly, the confusion matrix shows 90% recall for the extremely dense (FZ) class, meaning high sensitivity for the most hazardous condition in tunnel safety assessment. PCA visualization (Figure 17) confirms high inter-class separability, with three tight, disjoint clusters noted in low-dimensional embedding space.

The core innovation of ABRED lies not in statistical sophistication, but in structured knowledge injection. It explicitly encodes three domain-specific physical priors, including fracture multi-scale distribution, illumination multiplicative model, and local contrast invariance, into a lightweight, interpretable, and geometrically grounded feature representation. It thus achieves competitive accuracy with recent deep learning approaches on our dataset while operating at a fraction of the computational cost and data requirement and offering full transparency. It must be acknowledged that the reported competitiveness with deep learning methods is dataset specific. On our 1200-image tunnel dataset, ABRED (87.6%) performs comparably to recent CNN-based classifiers (typically 88–91% accuracy under similar training conditions). However, deep learning models are known to scale more favorably with data volume; with significantly larger annotated datasets (e.g., >10,000 images), CNNs are expected to surpass ABRED. Our contribution does not claim to replace deep learning, but rather to offer a practical, interpretable, and resource-efficient alternative for periodic safety monitoring in field operations where high-end GPU infrastructure is unavailable and model transparency is mandated by engineering safety regulations. We believe ABRED represents a practical, CPU-deployable solution for periodic tunnel monitoring systems, with potential for future optimization toward embedded real-time deployment.

More generally, ABRED exemplifies a22 paradigm shift for engineering vision tasks. With domain mechanisms (in this case, the mechanics of rock mass) known, explicit prior encoding—in lieu of end-to-end black box learning—can yield models that are simultaneously accurate, efficient, auditable, and physically meaningful. Each transformation from quadtree splitting to relative density normalization is interpretable visually, justifiable quantitatively, and directly interpretable in terms of geological interpretation, representing a “white box” benefit invaluable in safety-critical infrastructure applications.

(1) Rotation-invariant spatial partitioning: Existing axis-aligned quadtree segmentation breaks down upon image tilt (resulting in variable block layouts for the same fracture patterns). We will employ orientation-aware preprocessing—approximately obtaining dominant fracture direction using oriented gradient histograms—and either rotate images before partitioning or use rotation-robust super-pixel segmentation (e.g., SLIC with geodesic affinity) in place of the quadtree.
(2) Hybrid handcrafted–lightweight deep fusion: ABRED achieves high interpretability and efficiency, yet lags behind the deep hierarchies in semantics. Here, we attempt to close the gap by fusing of the ABRED features with the intermediate layer activations of pruned, quantized MobileNetV3, where white box transparency is preserved on low-level features, with an injection of high-level semantics and real-time inference on edge hardware with no compromise in accuracy.
(3) Granular, specification-aligned rock classification: The three-class scheme does not do justice to engineering practice, where support design relies on finer gradations (e.g., very sparse, moderately sparse, moderately dense). We will enrich the dataset with expert-annotated intermediate states and develop a five- or seven-grade classification framework directly mapped to national tunnel support standards to enable actionable, grade-specific excavation and reinforcement decisions.

## Figures and Tables

**Table 1:** Distribution of fracture image samples.

Fracture State	Code	Fracture Density Feature Description	Number of Samples
Sparse fractures	C4	Relatively intact rock mass, locally developed with a few joints, spacing > 4 cm, no penetrating joints	400
Dense fractures	C5	Fragmented rock mass, dense joints, spacing 2–4 cm, visible open joints	400
Extremely dense	FZ	Extremely fragmented, rock mass appears brecciated or muddy, fractures highly developed, often with signs of water seepage	400

**Table 2:** Main parameter settings of ABRED.

Parameter	Symbol	Value	Description
Minimum block size	$S_{min}$	8 pixel	Minimum side length for quadtree partitioning
Variance threshold coefficient	α	0.55	Ratio coefficient to global gradient variance
Canny low threshold ratio	$t_{low}/\mu_{M}$	0.4	Ratio relative to global gradient mean
Canny high threshold ratio	$t_{high}/\mu_{M}$	0.8	Ratio relative to global gradient mean
Canny Gaussian std	$\sigma_{Canny}$	1.0 pixel	Gaussian smoothing before edge detection
Relative density smoothing	ϵ	0.005	Small constant to prevent division by zero
Multi-scale Gaussian sigma	$\sigma_{s}$	{0.5, 1.0, 2.0}	Three smoothing scales for multi-scale extension
Single-scale feature vector max length (per-scale)	$K_{max}$	1024	Fixed dimension for zero-padding
SVM penalty parameter	C	1	Cost parameter for linear kernel SVM

Note: Classification task involves distinguishing three states: sparse (C4), dense (C5), and extremely dense (FZ).

**Table 3:** Accuracy of different methods.

Method	Accuracy (%)
FBD (Fixed + Absolute)	72.4 ± 1.3
FBR (Fixed + Relative)	75.1 ± 1.2
ABD (Adaptive + Absolute)	78.9 ± 1.1
LBP	79.2 ± 1.2
HOG	75.8 ± 1.4
Gabor	74.5 ± 1.3
ABRED (Proposed)	87.6 ± 0.9

**Table 4:** Accuracy degradation under different disturbances (%).

Disturbance	Gaussian Noise	Salt and Pepper Noise	Gamma Variation
FBD	−11.2	−13.5	−8.9
FBR	−9.8	−10.1	−4.2
ABD	−8.5	−7.3	−7.1
LBP	−7.2	−6.5	−3.3
HOG	−13.4	−11.2	−6.4
Gabor	−9.1	−7.8	−5.0
ABRED	−5.3	−4.8	−2.1

**Table 5:** Individual and combined effects of modules.

Blocking Method	Density Type	Sparse (C4)	Dense (C5)	Extremely Dense (FZ)	Overall
Fixed	Absolute	78.3	70.2	68.7	72.4
Fixed	Relative	80.1	73.5	71.7	75.1
Adaptive	Absolute	85.2	76.4	75.1	78.9
Adaptive	Relative	92.5	84.6	85.7	87.6

## Data Availability

The data presented in this study are available on request from the corresponding author. The data are not publicly available due to privacy or ethical restrictions.
